# Different conformations of the German shepherd dog breed affect its posture and movement

**DOI:** 10.1038/s41598-020-73550-x

**Published:** 2020-10-15

**Authors:** A. Humphries, A. F. Shaheen, C. B. Gómez Álvarez

**Affiliations:** 1 School of Veterinary Medicine, University of Surrey, Guildford, UK; 2 Department of Life Sciences, Brunel University London, London, UK; 3 Department of Veterinary Medicine, University of Cambridge, Cambridge, UK

**Keywords:** Animal physiology, Biomechanics

## Abstract

The conformation of the German shepherd dog (GSD) varies considerably within the breed. These differences may result in large variation in the movement and limb loading and undesirable consequences to their musculoskeletal health. This study aimed to investigate the relationship between conformation and biomechanical measures in 60 GSDs. Full body kinematic and kinetic measures were computed from 3D motion capture and pressure data. The dogs were divided into groups based on their back slope and curvature. Correlation analysis and statistical differences between groups showed that GSDs with a greater back slope have a greater contact area in their forelimbs and place them closer together when standing (n = 60). During trot, the dogs with sloped back showed a greater vertical force in the forelimbs and a greater mid-thoracic flexion (n = 60). Unilateral differences were found in the stifle flexion, hock flexion and hock adduction, suggesting greater movement asymmetry with an increase in the back slope (n = 30). In conclusion, several biomechanical parameters are affected by the GSD’s slope of the back and not by its curvature. Further studies are required to determine whether the variation in movement, posture and conformation within the breed relates to an increased susceptibility to musculoskeletal disorders.

## Introduction

Conformation varies enormously between dog breeds, even between large breeds of similar weight^1,2^. The different conformations are described in specific breed standards^2,3^, however, they tend to evolve with time. Currently, there are notable differences in the conformation of the purebred German shepherd dog (GSD), which is commonly observed as variation in the incline and curvature of the back and in the angulation of the hindlimbs. The modern purebred GSD is a breed that first started as a herding dog, and later as a working dog used by the police and the armed forces. Nowadays, there are two distinct types based on the dog’s activity; a working GSD and a showing GSD.

Despite little variation in the breed standard across the world, there are considerable differences in its interpretation by judges, breeders and dog’s owners; resulting in large variation in the breed, in particular their conformation, which can be categorised by the slope and curvature of the topline^2^. The current breed standard of the United Kingdom for the GSD^3^ states that “the topline (back) runs without any visible break from the set on of the neck … falling away slightly in a straight line to the gently sloping croup”. It is further stated that the croup should be “slightly sloping and without any break in the topline (and) merges imperceptibly with the set on of the tail”; where a flat or excessively inclined croup is undesirable^3^. The British breed standard of the GSD hindlimbs states that “Angulations (should be) corresponding approximately with front angulation, without over-angulation”, the standards further emphasise that over-angulation of the hindlimbs are undesirable^3^. However, despite this standard, a GSD is considered to have a favourable topline by many judges with an incline that progressively increases towards the croup, as this seems to be based on people’s perception of aesthetics of the breed rather than function and health. This perception has led to a profound change in the conformation of the breed over the last century, with the back profile evolving from relatively straight, rectangular conformation to a curved (Germanic type) and sloped conformation (British type)^4^. The various interpretations of the breed standard have led to a wide variety of conformations of GSD, with some conformations being perceived as having health related side effects and leading to unhealthy hindlimb mechanics in gait. For example, the Crufts 2016 show was hit with controversy and a media outcry when a GSD with a ‘sloping back’ won best in breed but appeared to be struggling to walk^5^. However, there is limited understanding of whether the evolution of conformation in the breed does indeed have significant effects on movement and if this relates to any clinical presentations.

Some studies have used kinematic and kinetic analysis of walk, trot and square standing to describe the gait and posture of specific canine breeds, such as the Labrador^6–8^, Doberman^9^ and Greyhound^6^, but not the GSD. When comparing the kinematics and kinetics of different breeds and between dogs of the same breed, it is important to consider if differences are due to variations in the way the dogs move or due to variation in body mass and size. After normalising kinetic parameters for size and body mass, Bertram et al.^6^ concluded that the movement and limb loading of the Labrador and Greyhound were dynamically similar during trot.

Other studies which measure the kinetics in dogs have used a pressure walkway^10–12^, force plates^8,13^ or weighing scales^14^ to record parameters such as limb loading, peak vertical forces, paw pressures and temporospatial (time and distance) parameters. This has shown that in normal stance, dogs support 60% of their weight on the forelimbs when standing square, although this value will change depending on the head and neck position^15^. The vertical forces and weight distribution during walk are also similar between breeds when normalised to the weight of the dog^10^.

Very few biomechanical studies have investigated the GSD. In a study with 10 healthy GSDs and 10 GSDs with hip dysplasia, GSDs with dysplastic hips were shown to have a greater angle of flexion at the hip and a greater rotational velocity of the hip when trotting^16^ and a lower peak vertical force compared to normal dogs^12,13^. These findings clearly indicate that the degree of hip dysplasia is related to the weight bearing and lameness in the GSD. Fischer and Lilje^2^ analysed the stride parameters and the angles of the joints in the sagittal plane in 10 working and 14 showing GSDs. They showed that the limb’s forward and backward range of motion during the stride was bigger in the show lines compared to the working lines, and that the show lines had a shorter femur and lower leg. This is probably the only attempt in the current literature to analyse the two breed lines with gait quantification methods, however the statistical significance of their findings was not reported. The kinetics and 3D kinematics of healthy GSDs across a range of conformations and types have not yet been fully investigated, hence it is not known if differences in conformation within the breed affects movement and standing posture and if this relates in any way to the risk of musculoskeletal disorders in this breed.

The present study aimed to quantitatively describe standing posture and trot of 60 healthy GSDs by analysing the kinematics and kinetics. The relationship between the kinematic and kinetic parameters and conformational measures of the back curvature and slope was investigated to determine if the conformation is likely to affect the standing posture and movement in the healthy GSD.

## Materials and methods

Sixty healthy, lameness-free, purebred GSDs of both sexes, any age from 6 months old, and any conformation type were recruited. All dogs had been previously hip scored and were eligible to participate if their hips were structurally sound [denoted by a hip score (HS) which was less than the mean for the breed (HS ≤ 16 from BVA/KC hip score statistics)]  (S2). Owners were sent a questionnaire to complete prior to the study, the questionnaire included information on their dog’s clinical history, activity levels, training, fitness and health. A copy of the questionnaire is included in the supplementary material S1.

### Ethics statement

All the methods have been approved by the institutional Ethical Committee from the University of Surrey’s (NASPA ethics committee). All protocols carried out in these animals were in accordance with relevant guidelines and regulations of this committee and the institution, and all owners gave written consent to participate in the study.

Dogs attended a single session in the Biomechanics Laboratory at the School of Veterinary Medicine in the University of Surrey. Dogs that showed any signs of anxiety or aggression (ASPCA SAFER behavioural assessment), were considered unfit to participate or were lame (following a general examination and visual gait assessment, respectively) were excluded from the study. Dogs were allowed some time to familiarise themselves with the biomechanics lab prior to data collection. This was followed by measurements of conformation and fitness. The conformation measures were as follows: the slope of the back calculated as the slope of the line connecting the withers and mid-sacrum to the horizontal and the length of the back (withers to mid-sacrum). To obtain these measures, a height measurement stick was used to measure the positions of the wither and mid-sacrum when the dogs were standing square and a tape measure was used to measure the length of the back. Indicators of the dog’s fitness were also recorded, these were: the body weight, muscle tone of the thighs (i.e. quadriceps muscles) and upper forelimb (triceps muscle) using a semi-objective scale based on palpation: (1) hypotonic, (2) normal tonicity and (3) hypertonic, body condition score (scale 1–9), mid-thigh circumference using a measuring tape calibrated for the handler’s pulling strength (GULICK II TAPE MEASURE MODEL 67020) and fat thickness using a fat calliper at the mid-lumbar region (S2). All measurements were obtained by the same observer^17^.

**Figure 1:**
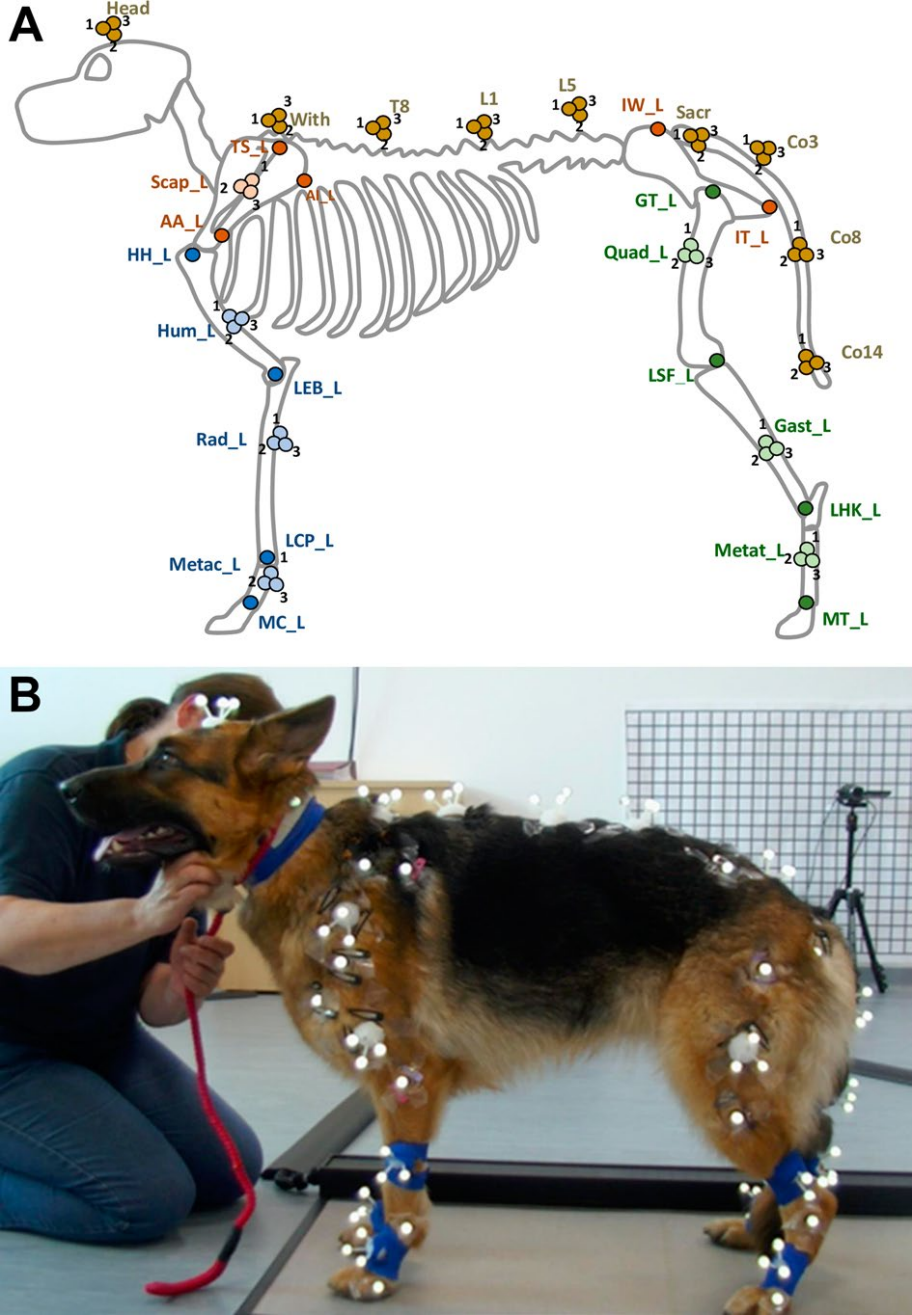
Illustration of reflective marker positions attached at bony landmarks and cluster locations on the left side and back (**A**). Markers were also attached at the same locations to the right limbs. Standing data collected while a GSD stood square on the pressure walkway for 10 s (**B**).

Reflective markers were attached to the dog’s skin using hypoallergenic double-sided tape; markers were placed at anatomical landmarks and clusters of reflective markers were attached to the middle of each segment, as shown in Fig. 1 and listed in the supplementary material (S3). A motion capture system consisting of eight infrared cameras (QUALISYS, Gothenburg) tracked the motion of the reflective markers in 3D space. This was synchronised with two video cameras and a two and a half metre high resolution pressure walkway (TEKSCAN, Biosense Medical, Chelmsford) which was used to measure the load distribution of each paw.

**Figure 2:**
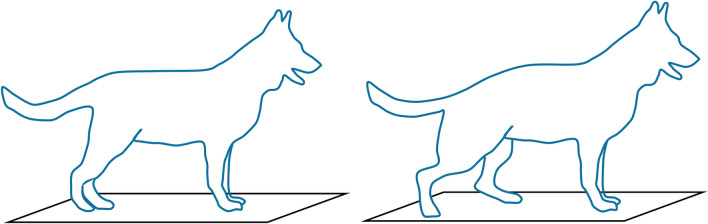
Diagram of the two standing postures studied: square (left) and stacking posture (right).

Kinematic and kinetic data were recorded while dogs stood square on the pressure walkway for 10 s; the paws were positioned so they were directly below the hip joint or humeral head (Fig. 1B). This was repeated three times and followed by three measurements while the dog stood in a stacking posture where their paws were positioned the same as during square standing, except their rear left limb was manually retracted until their lower hindlimb was vertical (Fig. 2, right). The stacking position is adopted in dog shows and some dogs (show dogs) were more comfortable adopting this position than square standing.

Dogs trotted over the pressure walkway at a comfortable, self-selected speed for twelve valid trot stride cycles; recordings were rejected if dogs were not looking ahead, tripped or changed speed. The tracked reflective markers were assigned for each dog to create a 3D model of the dogs and these were used to compute kinematic parameters in standing and trot.

**Figure 3:**
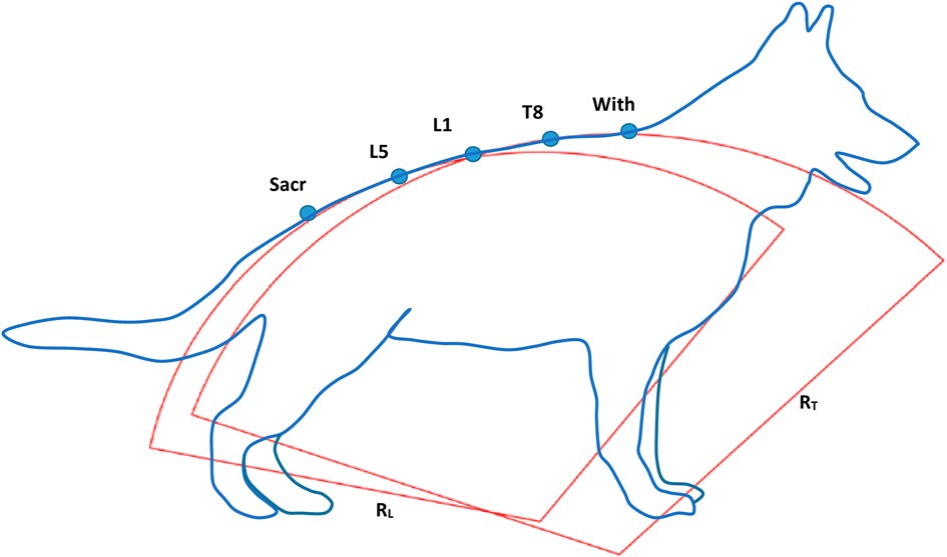
Illustration of the calculation of the radius of curvature of the thoracic (R_T_) and lumbar (R_L_) regions of the back using the location of the markers along the back (Sacr: sacrum, L5: lumbar 5, L1: lumbar 1, T8: thoracic 8, Wit: withers). Expressed in metres (m).

The centre of each cluster on the back was used to calculate the radius of curvature of the back in the thoracic (R_T_) and lumbar (R_L_) regions^18^ as shown in Fig. 3. Thus, dogs with a more arched back profile would have a smaller radius of curvature.

Marker positions were also used to describe the angulation of joints (flexion/extension, abduction/adduction and internal/external rotation) and limb positioning. The flexion/extension and abduction/adduction angles of each joint were calculated from the anatomical marker positions in the sagittal and transverse planes and axial rotation was quantified in VISUAL3D (C-MOTION, USA) using the clusters placed on the limbs and Euler sequence ZXY.

The protraction/retraction angles of the forelimb and hindlimb were also calculated for each limb, using the position of the metacarpal/metatarsal anatomical markers and the acromion/greater trochanter markers respectively. The data were filtered using a second order low pass Butterworth filter (with 6 Hz cut-off frequency) to minimise the errors associated with marker movement.

The markers placed at anatomical landmarks were also used to quantify the height of each segment above the ground (measured from the proximal end) and the segment length. Parameters were normalised to the dog’s height measured at the withers.

Trot stride cycles were split using the time of initial contact of the left forelimb recorded from the pressure mat. The maximum and minimum joint rotations and the range of motion for each joint were measured in four valid stride cycles for each dog and joint angles were plotted against cycle time.

Limb loadings, peak pressures, contact areas and weight distribution were quantified from kinetic data during standing and trotting; the loading of the digital pads and metacarpal/ metatarsal pads were also quantified for each limb to describe intra-paw variation. Data were normalised to body mass recorded from the pressure mat to ensure parameters were not affected by the weight of the dog. The stride length was normalised to the height at the withers to ensure that the size of the dog did not affect the findings.

### Statistical approach

Means of the kinematic and kinetic parameters were computed for each dog and used to calculate the intra and inter-subject standard deviations. All data recorded was tested for normality using the Shapiro–Wilk test and the slope and radius of curvature of the back were tested for independence using the Chi-squared test (see S2). As both parameters used to describe the back profile were independent (*p* = 0.371), the back slope and the curvature of the back were used to investigate relationship between the back profile and kinematic and kinetic parameters using linear and non-linear regression analysis, where appropriate.

**Figure 4:**
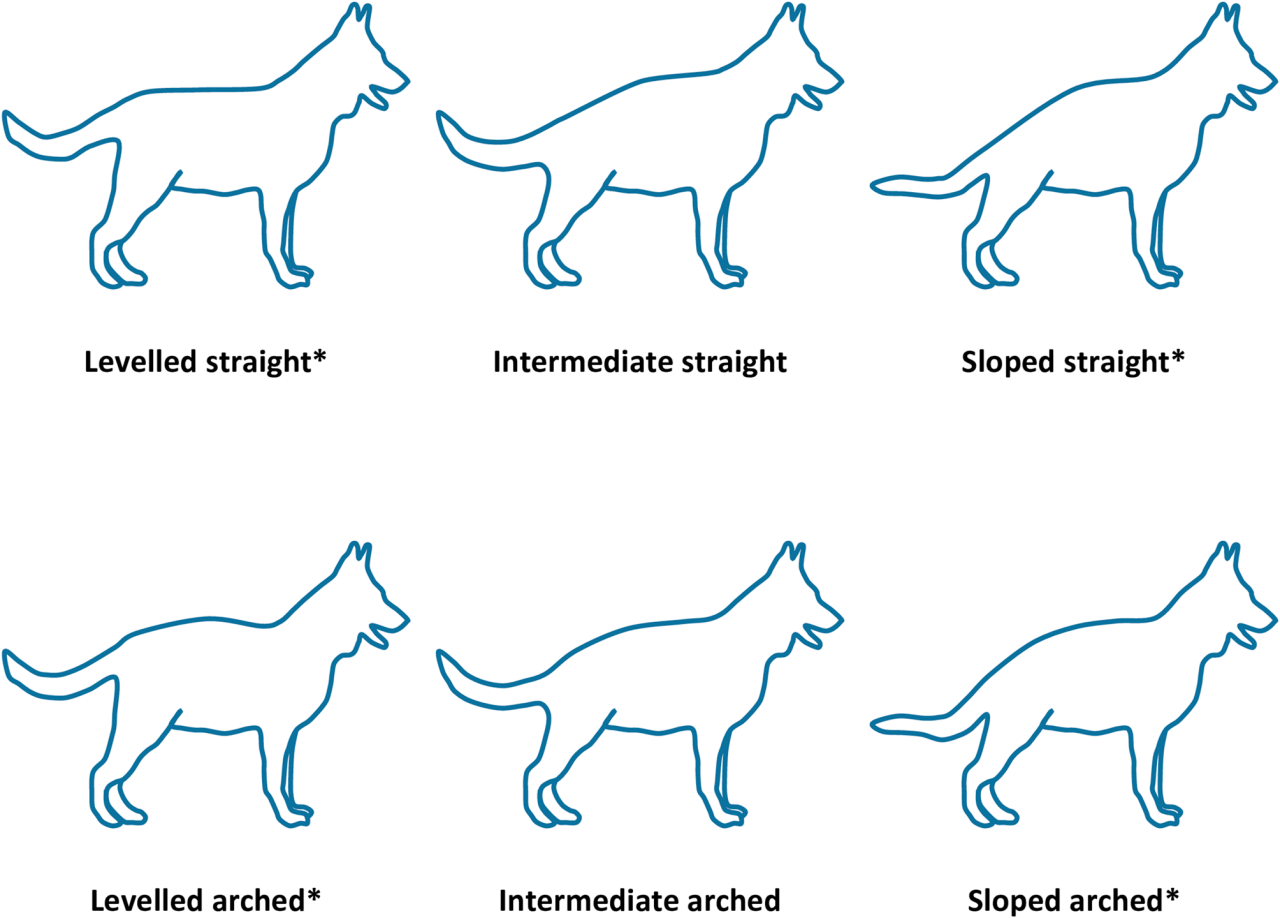
Schematic illustration of the GSD back profiles: straight and arched curvatures; and: levelled, intermediate and sloped inclinations. *Modified from Fischer and Lilje^2^ and Winfrow^4^.

The back slope and thoracic radius of curvature (R^T^) were also used to assign each dog to a theoretical conformation group (levelled back: < 3°, intermediate back: 3°–6°, sloped back: > 6°) and curvature group (arched back profile: R_T_ < 1 m, straight back profile: R_T_ > 1 m) (Fig. 4). Chi-square tests were used to test differences in the groups in nominal variables: sex, percentage in agility and participation in shows, posthoc tests with Bonferroni correction was used when differences were found. For continuous variables, Kruskal–Wallis tests with Dunn’s posthoc analysis (*p *values adjusted for multiple comparisons) were used to check for differences between the groups. To avoid type 1 errors, biomechanical measures were only considered to be affected by conformational measures if (both) significant Pearson’s correlations with the conformational measure (back slope or curvature) and differences between the conformational groups were found. Finally, Freidman tests were used to test for differences between standing positions (square and stacked).

## Results

A total of 60 (31 females) GSDs were recruited in the study with a mean age of 4 years and 2 months (SD: 2 year and 3 months), mean body mass of 39.8 kg (SD: 7.5 kg) and a mean combined hip score of 8.1 (SD: 3.8). Back inclinations ranged from 0° to 10.34° (mean: 5.0°, SD: 2.7°) and the mean radius of curvature of the thoracic region of the back was 2.26 m (SD: 4.00 m). When categorised by slope, 18 dogs were in the levelled back group, 21 in the intermediate and 21 in the sloped back group. When categorised by curvature of the back, 29 dogs were in the arched back profile group and 31 in the straight back profile group. In total, 38% of the GSDs were trained in agility and 42% of dogs had participated in breed shows; in particular, most GSDs in the sloped back group (68%) participated in dog shows, whereas 17% of GSDs in the levelled back group participated in dog shows (*p* = 0.006). The height of the sacrum was significantly higher in the levelled group than in the intermediate (*p* = 0.012) and sloped (*p* < 0.001) groups. Dogs with levelled backs had a greater thoracic radius of curvature compared to dogs in the sloped back group (*p* = 0.017).

In keeping with the statistical approach, kinetic and kinematic parameters related to conformational measures are only reported here if they were found to be significantly correlated to the conformational measure (back slope or curvature) and differences between conformational groups were also found. The results showed that a number of kinetic and kinematic parameters were related to the back slope (detailed below) but none were shown to be affected by the curvature. The full results of the statistical tests are shown in supplemental material (S2–S7).

It was not always possible to acquire full 3D kinematics of the 60 dogs because of obstructions to the marker trajectory by the owner on one side of the dog; this was particularly the case for forelimb joints kinematics in trot. In addition, the quality of data were compromised in some occasions due to the dogs’ fur. As a result, all the figures depicting kinematic results have been limited to high-quality data obtained from 30 dogs.

### Standing posture

In standing, the main kinetic parameter affected by the slope of the back was the contact area of the forelimbs which was correlated with the slope of the back during both standing positions (square: correlation coefficient (r) = left 0.37, right 0.44, *p* < 0.01; stacked: r = left 0.44, right 0.45, *p* < 0.001); GSDs in the sloped back group had a greater contact area than dogs in the levelled back group (square: right *p* < 0.001 left *p* = 0.006, Stacked: right and left *p* = 0.002). Additionally, in the stacked position, the percentage of weight bearing on the forelimbs was correlated with the slope of the back and inversely correlated in the hindlimbs (r = 0.33, *p* = 0.010).

In the hindlimb, the angle of retraction was correlated with the slope of the back during square standing (right: r = 0.51 and left 0.56, *p* < 0.001). Dogs with a levelled back had a mean retraction angle of 6.10° (SD: 2.19°) in the left and 4.68° (SD: 2.96°) in the right hindlimb and dogs with a sloped back had a greater retraction angle of 10.71° (SD: 5.26°) in the left (*p* = 0.003) and 10.45° (SD: 6.12°) in the right hindlimb (*p* = 0.002). The abduction/adduction of the left and right hindlimb joints were not found to be (consistently) affected by the slope of the back.

The forelimbs were positioned closer together during both standing positions in dogs with a greater slope of the back (expressed as a percentage of wither’s height) (square: r = 0.58, *p* < 0.001; stacked: r = 0.60, *p* < 0.001). When comparing groups in square standing, GSDs in the levelled back group positioned their forelimbs with a distance of 40.2% (SD: 3.8%) of the wither’s height, whereas GSDs in the sloped back group positioned their forelimbs closer, at a distance of 34.0% (SD: 4.1%) of the wither’s height (*p* < 0.001). The flexion of the thoraco-lumbar joint was also found to be affected by the back slope in the two standing positions, where a greater thoraco-lumbar flexion was found is dogs with a greater back slope (square: r = 0.043, *p* = 0.001; stacked: r = 0.34, *p* = 0.007).

**Figure 5:**
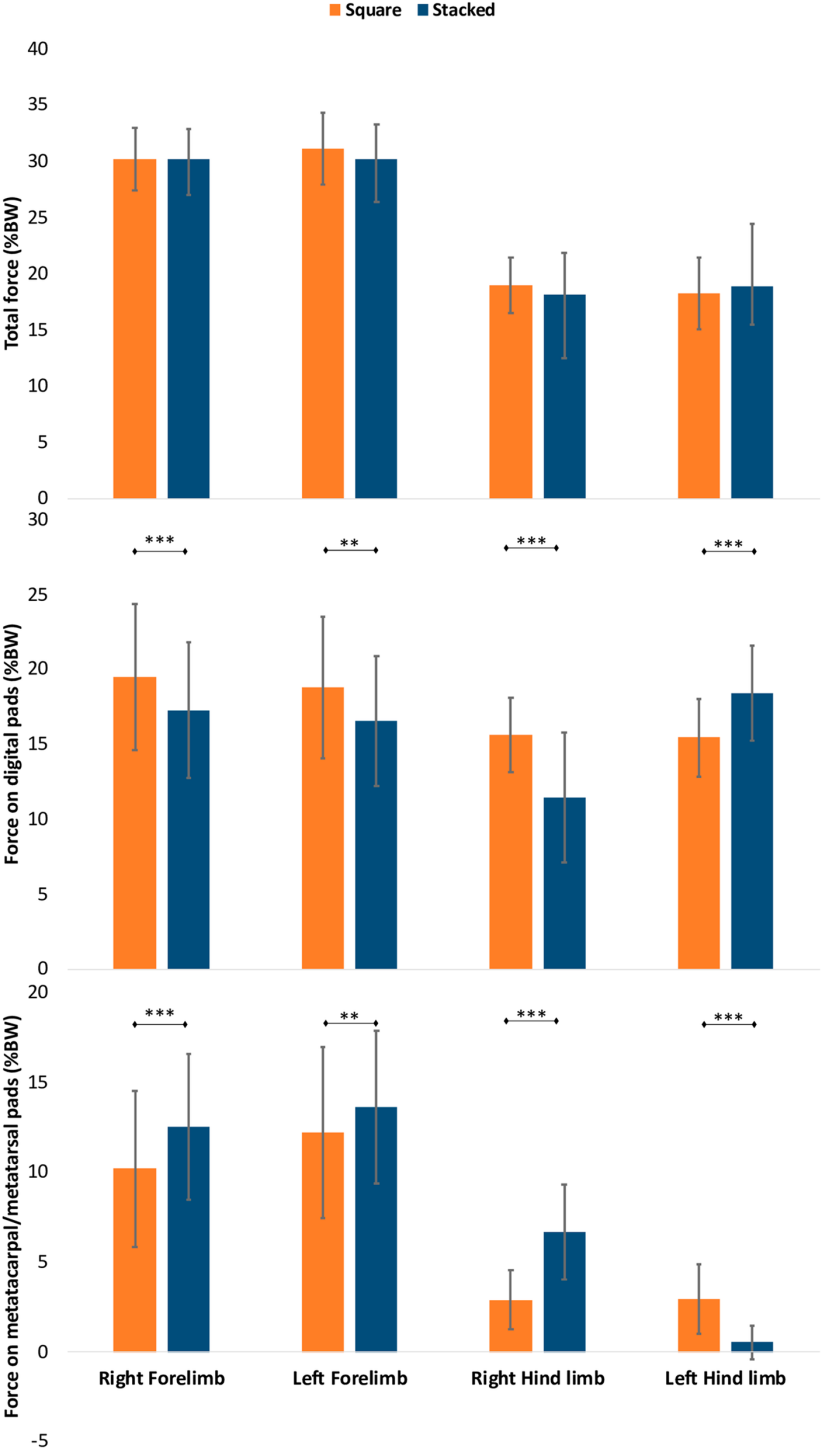
Vertical force supported by the limbs, digital pads, metacarpal/metatarsal pads of each limb when standing square and in the stacked position expressed as a percentage of body weight (BW), showing the mean and standard deviation for the 60 GSDs. Significant differences are indicated using *For *p* < 0.05, **for *p* < 0.01, **for *p* < 0.001.

There was no difference in the limb loadings between both standing positions and these were symmetrical between the left and right. However, there were differences in the loading of the metatarsal and digital pads in each paw. In the stacked position, dogs carried a greater percentage of their body mass unretracted hindlimb, the weight was transferred towards the metatarsal pad and away from the digital pads when standing in the stacked position (11.5%, SD: 4.3%) compared to the loading in the digital pads when standing square (15.6%, SD: 2.5%) (*p* < 0.001) (Fig. 5). Furthermore, the load in the forelimbs was supported more by the digital pads during square standing compared to the stacked position (right *p* < 0.001, left *p* = 0.005) (Fig. 5 and S4).

**Figure 6:**
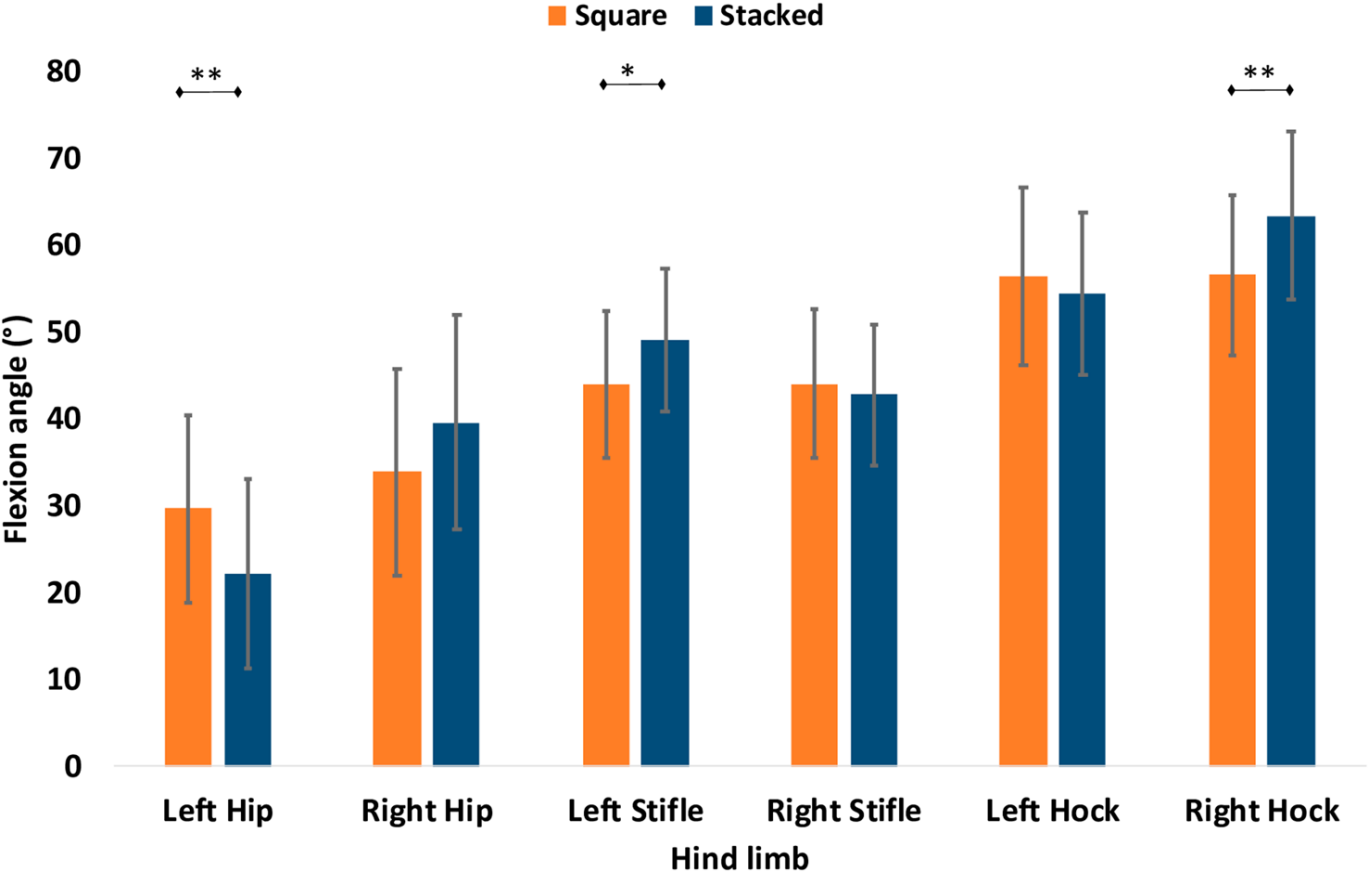
The flexion angle of the hindlimb joints when the GSD stands square and in the stacking position with the left hindlimb retracted, showing the mean and standard variation for the 60 GSDs. Significant differences are indicated using *For *p* < 0.05, **for *p* < 0.01, **For *p* < 0.001.

Comparing the postural kinematics of the two standing positions, the GSD retracted their left hindlimb with a mean retraction angle of 22.81° (SD: 4.36°) and protracted their right hindlimb (− 4.04°, SD: 5.71°), whereas during square standing the left hindlimb was retracted by 8.55° (SD: 4.25°) and the right hindlimb by 8.30° (SD: 5.07°). To stand in the stacked position, GSDs extended their left hip (*p* = 0.001) and increased the flexion of the left stifle (*p* = 0.035) while retracting the left hindlimb. The flexion of the hock in the retracted limb did not change compared to standing square, but in the opposite hindlimb the hock joint became more flexed (*p* = 0.005) (Fig. 6).

### Trot

In trot, the swing phase duration of the hindlimbs were correlated with the slope of the back (right r = 0.34, *p* = 0.007; left r = 0.27, *p* = 0.034), where dogs in the sloped back group had longer swing times and shorter stance times (58:42) than dogs in the levelled back group (56:44); interestingly, this difference did not lead to a change in speed.

The forelimbs of dogs with a greater back slope had greater vertical force during trot (right r = 0.58, left r = 0.55, *p* < 0.001). GSDs in the sloped back group had a mean vertical force in the forelimbs of 128.6% of the body weight (SD: 17.6%), which was greater than in the levelled back group with a mean of 106.9% of the body weight (SD: 11.6%), (*p* < 0.001). Loading of the digital pads was greater in both forelimbs (right *p* = 0.001, left *p* = 0.022) and both hindlimbs (right and left *p* < 0.001) in GSDs with a more sloped back, but the loading of the metacarpal and metatarsal pads were not consistently affected by the slope of the back (S6).

**Figure 7:**
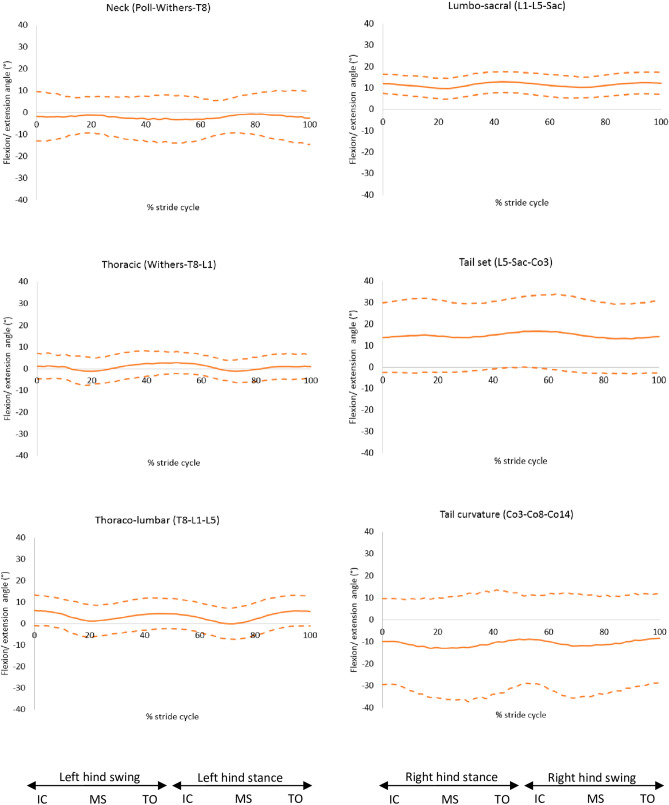
Flexion (positive) and extension (negative) angles of the back segments, mean of 30 GSDs (solid line) and showing one standard deviation from the mean (dashed lines). Graphs are plotted for one stride cycle, showing initial contact (IC), Mid Stance (MS) and Toe Off (TO) for the left (right hind stance/left hind swing) and right diagonals (left hind stance/right hind swing). Between brackets: marker names (Supplementary material 3, S3).

Results from the kinematic data showed that the mid-thoracic region was more flexed in dogs with a more sloped back (r = 0.38, *p* = 0.003). GSDs in the sloped back group had a mean flexion angle of 7.9° (SD: 3.4°), which was greater than the levelled back group with a mean of 3.6° (SD: 2.4°). The flexion angles of the other regions of the back were similar for all GSDs (S7), these can be seen in Fig. 7.

**Figure 8:**
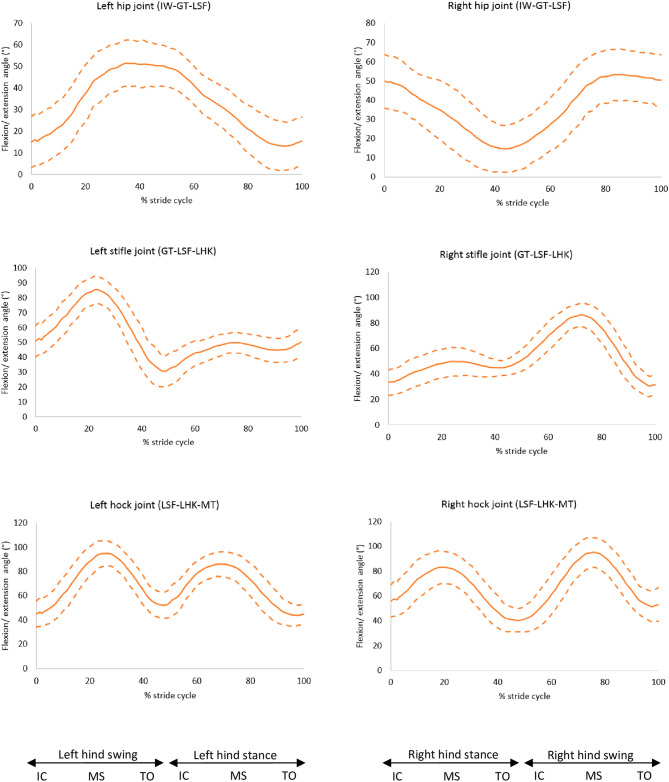
Flexion (positive) and extension (negative) angles for the hindlimb joints, mean of 30 GSDs (solid line) and showing one standard deviation from the mean (dashed lines). Graphs are plotted for one stride cycle, showing initial contact (IC), Mid Stance (MS) and Toe Off (TO) for the left and right diagonals. Between brackets: marker names (Supplementary material 3, S3).

**Figure 9:**
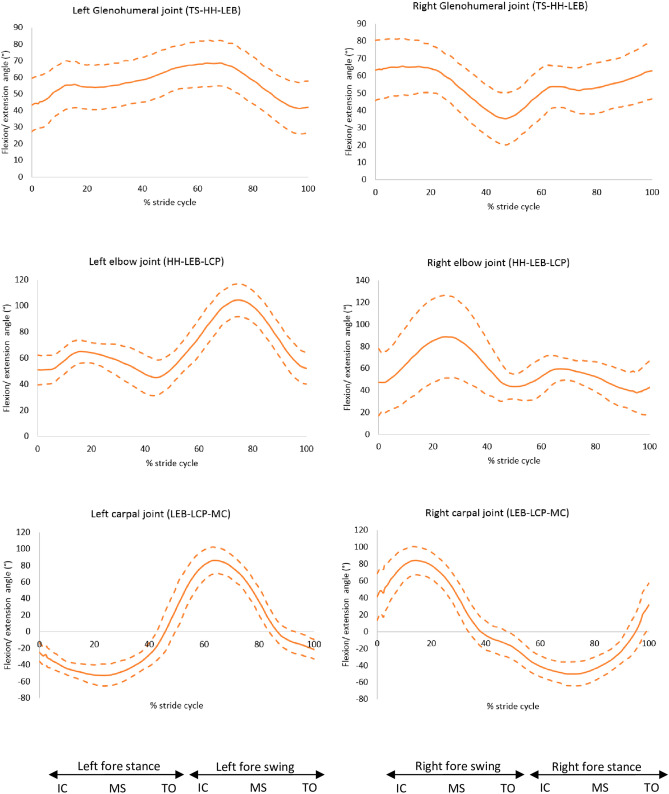
Flexion (positive) and extension (negative) angles for the forelimb joints, mean of 30 GSDs (solid line) and showing one standard deviation from the mean (dashed lines). Graphs are plotted for one stride cycle, showing initial contact (IC), Mid Stance (MS) and Toe Off (TO) for the left and right diagonals. Between brackets: marker names (Supplementary material 3, S3).

The flexion of the right stifle in trot was correlated with the slope of the back (r = 0.36, *p* = 0.005). GSDs in the sloped back group had a mean flexion angle of 94.1° (SD: 7.9°) which was greater than the levelled back group (mean: 85.0°, SD: 5.6°) (*p* = 0.012). The left hock remained more flexed throughout the trot stride cycle in dogs with a greater back slope (r = 0.31, *p* = 0.017), where dogs in the sloped back group had a mean minimum flexion angle of 38.6° (SD: 7.0°) which was greater than the levelled back group (mean: 33.7°, SD: 3.8°) (*p* = 0.028). The flexion/ extension angles of the other joints were not affected by the slope of the back (Figs. 8, 9, and S7).

**Figure 10:**
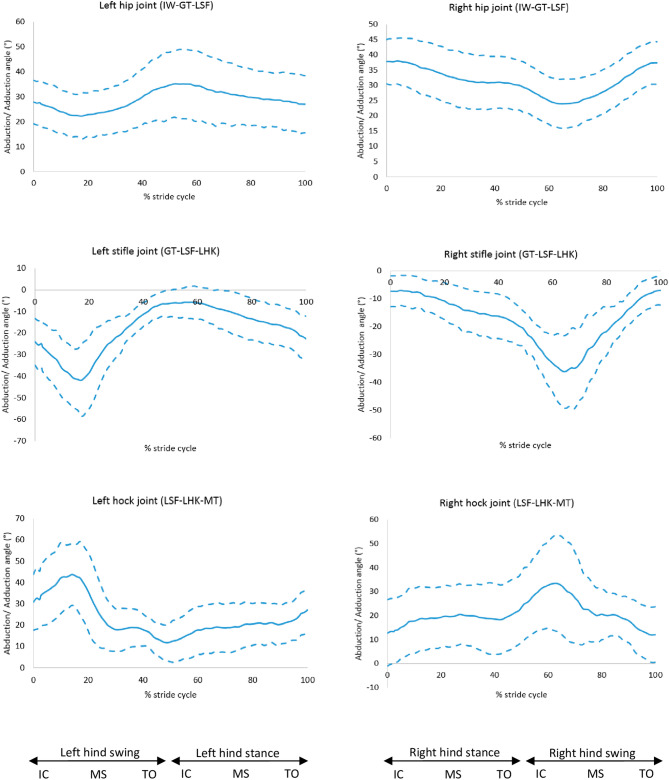
Abduction (positive) and adduction (negative) angles for the hindlimb joints, mean of 30 GSDs (solid line) and showing one standard deviation from the mean (dashed lines). Graphs are plotted for one stride cycle, showing initial contact (IC), Mid Stance (MS) and Toe Off (TO) for the left and right diagonals. The three reflective markers tracked and used to compute the joint angle is shown in the brackets. Between brackets: marker names (Supplementary material 3, S3).

The right hock was more adducted in GSDs with a greater back slope (r = 0.56, *p* = 0.001). The right hock of GSDs in the levelled back group had a greater abduction angle (mean: 21.2°, SD: 11.2°) compared to the sloped back group (mean: 0.5°, SD: 7.3°), (*p* = 0.001). The abduction/adduction angles of the other joints were not affected by the slope of the back (Fig. 10 and S7).

**Figure 11:**
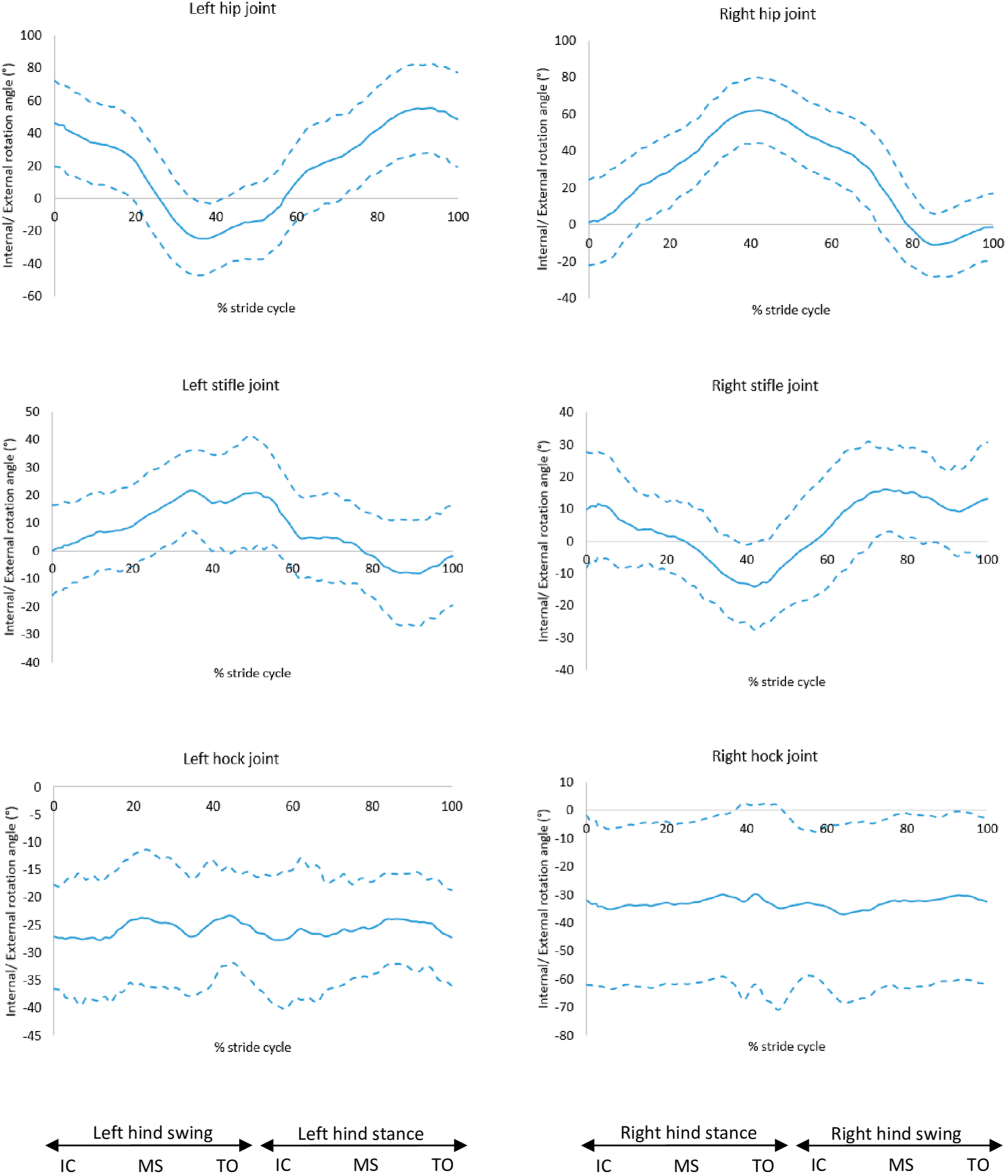
Internal (positive) and external (negative) rotation for the hindlimb joints, mean of 30 GSDs (solid line) and showing one standard deviation from the mean (dashed lines). Graphs are plotted for one stride cycle, showing initial contact (IC), Mid Stance (MS) and Toe Off (TO) for the left and right diagonals.

The internal and external rotation of the joints showed no correlation with the slope of the back. However, the results showed that in all GSDs the hips were internally rotated during the initial swing phase of the limb and during the stance phase, with a mean maximum internal rotation of 63.3° (SD: 22.9°) for the left hip and 67.1° (SD: 16.7°) for the right hip. During the swing phase, the hips were externally rotated, as shown in Fig. 9. The hocks were shown to be externally rotated throughout the stride cycle, where the mean maximum external rotation for the left hock in all the GSDs was 40.4° (SD: 10.3°) and for the right hock was 39.7° (SD: 9.1°) (Fig. 11 and S7).

**Figure 12:**
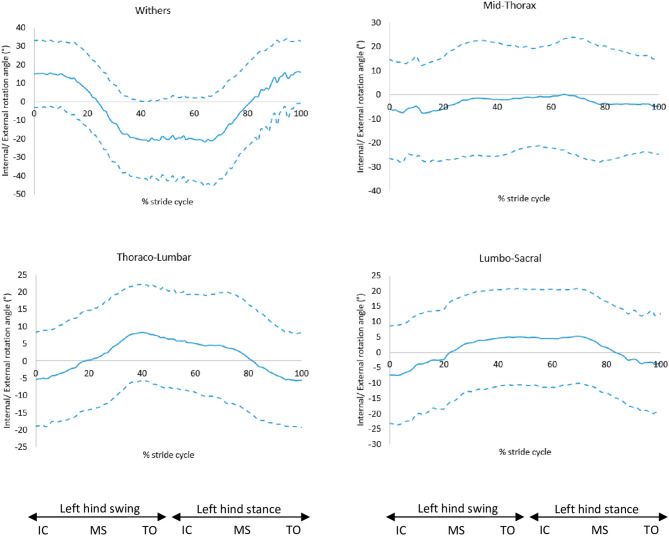
Right (positive) and left (negative) rotation for the back segments, mean of 30 GSDs (solid line) and showing one standard deviation from the mean (dashed lines). Graphs are plotted for one stride cycle, showing initial contact (IC), Mid Stance (MS) and Toe Off (TO) for the left and right diagonals.

The slope of the back had no significant effect on the internal or external rotation of the back during trot. The thoraco-lumbar and lumbo-sacral regions of the back were axially rotated to the right side of the dog (positive) during the late swing phase of the right front limb and left hindlimb, as these limbs are lowered to the ground; whereas, towards the end of stance phase, these axially rotated to the left (negative) (Fig. 12). Conversely, the opposite is seen for the withers, as the region is rotated to the left side during late swing and early stance of the left hindlimb, and rotated to the right towards the end of the stance phase (Fig. 12 and S7).

## Discussion

This study gives detailed quantification of the three-dimensional movement during trot, standing posture and limb loadings of GSDs of a range of conformations; and provides evidence that these are affected by the slope of the back. Each parameter was tested for correlations with measures of conformation including the slope of the back and its curvature. In addition, dogs were categorised into groups according to their back slope and back curvature. The results showed correlations between kinetic and kinematic measures with back slope. Conversely, the curvature did not have an effect on any of the biomechanical measures.

Due to differences in fur, the quality of the kinematic data in the trot trials in some dogs were compromised. Therefore, to ensure the trot kinematic data were reliable and repeatable, a representative sample of the best 30 GSDs data were presented during trot. This did not affect the reliability of the standing kinematic data or in the kinetic data. Besides of problems with the fur, skin displacement alone is reported to affect non-sagittal motion analysis in dogs^19^. Therefore, care must be taken when making conclusions related to mediolateral and transverse plane rotations. Findings showed inconsistencies in the effect of back slope on some kinematic measures between the right and the left, for example, back slope was shown to be correlated with an increase in the right stifle flexion, the left hock flexion and right hock adduction. These results did not reach statistical significance on the contralateral side. To ensure that these results are real, we have visually assessed the synchronised video recordings for confirmation and indeed more GSDs with sloped backs presented an irregular motion pattern of one or both sides when trotting compared with the more levelled back dogs which is likely to be a cause of the inconsistencies found between the two sides.

The increased loading of the forelimbs in dogs with a greater back slope suggests that sloped dogs were leaning forward, thus increasing their forelimb weight bearing. This agrees with the observations by Hollenbeck^20^ indicating that GSDs naturally stand with their forelimbs well under the forechest. Other breeds carry approximately 60% of their weight in their forelimbs and 40% in the hindlimbs^2^ during normal standing, where this percentage can increase by 10–15% if the dog extends or lowers the head and neck^15^. Furthermore, in data analysed by the researchers on 12 sound Labrador retrievers, it was found that they carry 69% (SD: 5%) of their weight in the forelimbs, which is higher than the sloped back GSDs of the present study in spite of being both similar sized breeds. This means that although GSDs with a sloped back have an increased loading of the forelimbs, the loading distribution is comparable to other breeds and is still less than in a similar large breed. It can be speculated that this can either lead to, or, be due to more well-muscled forelimbs compared to the musculature in the hind quarters of sloped back GSD. Although this study recorded the muscle tone on the forelimbs and hindlimbs and the circumference of the thighs as indirect measures of muscle development and strength of the GSDs, there was no difference between the groups. Although it should be noted that these are semi-quantitative analyses that might not be very precise, hence further conclusions on differences in musculature and its relation to back slope in GSDs cannot be made from this study alone.

In both standing positions, square and stacked, the greater contact area of the forelimbs in dogs with a sloped back was independent of the increased weight bearing. When dogs stood in the stacked position, there was no difference in the loading of the forelimbs and hindlimbs compared to standing square, which means that dogs were able to maintain a symmetrical weight bearing in both standing postures. In order to achieve this during stacking, the dogs extended the hip and flexed the stifle of the retracted limb and loaded the paw mainly through the digital pads; whereas the limb which was not retracted had a more flexed hock to transfer more of the weight onto the metatarsal pad. Although the stacked position seemed more difficult to achieve for a few dogs, particularly those not trained for shows, their limbs were not loaded differently, suggesting no disadvantage or discomfort when standing in either position. Dogs with a more inclined back also positioned their forelimbs significantly closer together during standing, and this was not due to a difference in the widths of the thorax, defined in this study by the distance between the scapulae.

Flexion of the mid-thoracic region of the back was the only spinal region significantly correlated to back slope at trot. In most of the GSDs with straight back profile, the greatest flexion initiated at the thoraco-lumbar and lumbo-sacral regions, whilst dogs with an arched back profile had a curve originating from the thoraco-lumbar region. In an in vitro study by Benninger et al.^21^, the vertebral columns of 9 GSDs was found to have less mobility at L7–S1 than in dogs of other breeds, it was suggested that these findings were related to the detailed anatomy of vertebral facets in this breed. Further in vivo studies would be necessary to compare spinal motion with other breeds, in particular of the caudal spine, as the morphology and morphometry of the lumbosacral region has been assumed to promote lumbosacral disease in the GSD^22^.

The slope of the back was significantly correlated with increased values of several angle rotations of joints of one or the other hindlimb suggesting asymmetry of the hindlimb motion. Bockstahler et al.^23^ found that Belgian shepherd dogs (BSDs) with borderline hip dysplasia had a more flexed stifle joint compared to healthy BSDs and Miqueleto et al.^16^ reported greater flexion angles in GSDs with hip dysplasia. However, the current study showed no correlation between the back slope and the maximum hip flexion angles. Although it should be noted that dogs in this study were only included if they had hip scores less than the mean of the breed and therefore all dogs in this study were cleared from hip dysplasia. The inclusion criteria served to ensure that dogs included in this study were not subclinically lame. However, it may be argued that such a criterion biases against certain conformations. Anecdotal evidence from dogs recruitment suggests that dogs with more sloped backs were more difficult to recruit. Nevertheless, despite having this criterion dogs included in the study had a large range of back slopes and back curvatures which were the two conformational measures of interest.

During trot, there was a greater adduction angle and greater range of motion (ROM) of one of the hocks in dogs with a sloped back (i.e. ‘cow hock’), which is undesirable in the GSD breed, as it can be considered as broken columns of limb support, however it appears to provide lateral stability^20^. These findings might be linked as compensatory mechanisms to maintain a better balance, however, non-sagittal rotations cannot be directly attributed to biological reasons considering the great latero-medial skin displacement reported in the literature for the hindlimb in dogs^19^. It is still unclear why the GSDs with a more sloped back show more variable right-left sagittal plane movements of the stifles and hocks. The relevance of this findings might be important for the GSD’s musculoskeletal health, or at least, it might explain why some sloped back dogs seem to move more irregularly in the show ring without presenting explicit lameness.

The higher loading of the forelimbs and lower of the hindlimbs of sloped GSDs during trot and stacked standing suggests that this could lend the forelimbs a greater chance to exercise and develop musculature, while the reduced use of the hindlimbs in comparison could be a contributing factor to GSDs being perceived as having weaker hindquarters. Previous studies have found that GSDs with hip dysplasia also have a lower peak vertical force in their hindlimbs compared to normal dogs^12,13^, suggesting the reduced vertical force in the hindlimbs during standing and trot may be related to the development of dysplasia of the hip.

During trot, the increased loading of the digital pads in all limbs in sloped GSDs would normally be attributed to differences in speed^6^, however, all dogs moved at a comparable self-selected trot speed. Therefore, most likely, the differences in loading are likely to be related to differences in body conformation^6^. More levelled backed dogs distributed the loading more evenly between digital pads and metacarpal and metatarsal pads, however, neither of these ways of loading the limbs seem to have a clear advantage or disadvantage.

In our study, the slope of the back did not affect the segments’ lengths or the protraction/ retraction angles significantly, which disagrees with the study performed by Fischer and Lilje^2^ in 10 working and 14 showing GSDs where they found that limb’s forward and backward range of motion during the stride was bigger in the show lines compared to the working lines, and that the show lines have a shorter femur and lower leg. The authors did not report the back slope of their dogs, which could have been different than in our study. Other anatomical differences may have been present in their dogs since they were recruited in Germany and it is known that morphological differences between the Germanic type and British type exist^4^.

When comparing with other large breeds reported by Fu et al.^24^, the GSD had a more extended hip joint by approximately 20°, more flexed stifle by approximately 20° and a greater maximum flexion angle of the hock of approximately 38° during trot. Conversely, the GSD had comparable flexion and extension angles of the forelimb to other larger breeds^24,25^. These differences emphasise how the kinematics can be directly affected by the conformation of each breed.

All dogs internally rotated their hip joints for the majority of the stride cycle, only showing external rotation at the end of the swing phase. However, the hips of other larger breeds were externally rotated throughout the stride cycle, with a mean range of motion of axial rotation from 24° external rotation to 7° external rotation^24^, with the greatest amplitude of external rotation occurring at the end of the swing phase. Furthermore, the hocks were more externally rotated in the GSDs of this study compared to other larger breeds with a mean range of motion of axial rotation from 13° external rotation to 5° external rotation^24^; the GSDs also had considerably greater inter-subject variation in the hock axial rotation angle. The externally rotated hocks may be a compensating mechanism for the internally rotated hips. It is widely known that GSDs tend to present externally rotated hocks, in which case the metatarsus are externally rotated in relation to the tibia, resulting in the lower limbs rotating outwards, a feature of some dogs presenting the so called ‘cow hocks’. Hollenbeck^20^ explains that lack of muscle balance in the hindlimbs in dogs with a spread stance can result in cow hocks. This suggests that maintaining a healthy muscle mass in the hindlimbs can be essential in this breed with tendency to rotate hindlimbs externally.

An extensive range of kinetic and kinematic parameters were analysed in this study. However, parameters were only reported in the results if they were correlated with the slope of the back in addition to showing differences between the back slope groups, as this meant the parameters had a statistically significant and relevant relationship with the slope of the back. This statistical approach ensures that the reported results are truly significant and any possibility of randomness is discarded.

In conclusion, this comprehensive description of limb loading and joint angles of the GSD shows that this breed moves and stands differently compared to other breeds; and when comparing different lines within the breed as a result of variation in conformation, particularly the inclination of the back and not by its curvature or other conformation features. GSDs with sloped back carry more load in their forelimbs during standing and trot, and load their digital pads in all limbs more than the metacarpal and metatarsal pads; they also showed greater right-left differences in their hindlimb movement, particularly in the stifles and hocks. Although there are clear differences in standing posture and movement that are a result of changes in conformation, further research is needed to investigate whether the observed biomechanical changes are also related to the prevalence of musculoskeletal disorders in the GSD.

## Supplementary information

Supplementary Information 1.

Supplementary Information 2.

## Data Availability

The authors confirm that derived data supporting the findings of this work is available within the manuscript and the supplementary files. Raw data supporting the findings of this work is available from the corresponding author upon reasonable request.
