# Supplementary material for: Different conformations of the German shepherd dog breed affect its posture and movement
Source: Sci Rep. 2020 Oct 15;10:16924. doi: 10.1038/s41598-020-73550-x (PMC7567065; doi:10.1038/s41598-020-73550-x)
Supplement: Supplementary file 1 — Supplementary Information 1. [file 41598_2020_73550_MOESM1_ESM.doc]

Different conformations of the German shepherd dog breed affect its posture and movement

A Humphries^1^, AF Shaheen^1,2^, CB Gomez Alvarez^1, 2, 3*^

^1^ School of Veterinary Medicine, University of Surrey, Guildford, UK; ^2^ Department of Life Sciences, Brunel University London, UK; ^3^  Department of Veterinary Medicine, University of Cambridge, UK

* corresponding author= Constanza Gomez Alvarez [c.gomezalvarez@surrey.ac.uk](mailto:c.gomezalvarez@surrey.ac.uk)

Supplementary material:

Study ID:

**S1: Questionnaire**

**Questionnaire**

**‘Assessing the relationships between Conformation, Gait and Health and Welfare in Canine Breeds: German Shepherd Dog’**

Please answer the questions below, giving your contact details and your dog’s clinical history and activity levels. More space is available at the end of the questionnaire if required.

All information you provide will remain confidential and your personal details will not be disclosed at any time. The questionnaire will only be accessible to the research team and your details and responses in the questionnaire will not be shared with third parties.

| **Question 1: Your dog’s details** | |
| --- | --- |
| Dog’s name |  |
| Age (Years, months) |  |
| Breed |  |
| Colour |  |
| Sex |  |
| Neutered | Y / N |
| Hip score |  |
| Elbow score |  |
|  |  |
| **Question 2: Your contact details** | |
| Name |  |
| Phone number |  |
| Email address |  |

| **Question 3: Your dog’s clinical history** | |
| --- | --- |
| 3.1 | Is your dog currently suffering from any medical condition? If Yes, please provide details (such as: The name of the condition, the length of time your dog has had the condition and any treatment your dog is receiving). |
|  |  |
|  |  |
|  |  |
|  |  |
|  |  |
| 3.2 | Has your dog previously had any medical conditions? If Yes, please describe the type of condition. |
|  |  |
|  |  |
|  |  |
|  |  |
|  |  |
| 3.3 | Has your dog previously had surgery? If Yes, please provide details (such as: The reason and when your dog had the surgery). |
|  |  |
|  |  |
|  |  |
|  |  |
|  |  |
| 3.4 | Has your dog ever been diagnosed as overweight or obese? If Yes, please state when this was. |
|  |  |
|  |  |
|  |  |

| **Question 4: Your dog’s activity and behavioural levels** |
| --- |

In the following questions, please place a vertical line on the scale to indicate your response. Please not the labels for each of the scales.

| 4.1 | Over the last month, my dog’s attitude has been:  Negative  Positive |
| --- | --- |
| 4.2 | My dog engages in playful activities (such as playing with a ball or fetching a stick):  Rarely  Very often  *Please describe some further details of your dog’s playful activities:*   | Please select all playful activities your dog enjoys:  Ball games: , Retrieving/ fetching games: , Tugging/ pulling games: | | --- | | Are there other dogs in the house for your dog to play with? If so, how many? | |
| 4.3 | My dog is willing to play voluntarily:  Not at all  Very willing |
| 4.4 | My dog gets voluntary exercise:  Rarely  Frequently |
| 4.5 | Each day, my dog gets exercise (including walks):  Less than once  All day  *Please describe some further details of your dog’s exercise (including walks):*   | How many times a week your dog get exercise? | to a week | | --- | --- | | How many times a week does your dog exercise on lead? | to a week | | How many times a week does your dog exercise off lead? | to a week | | How long does each exercise last (in minutes)? | to minutes | | Over what type of terrain does your dog exercise? | Forest/grass  Pavement/street | |
| 4.6 | To get into the car, my dog:  Must be picked up  Can jump easily |
| 4.7 | To lie down, my dog finds it:  Difficult  Easy |
| 4.8 | To sit on his/ her haunches, my dog finds it:  Difficult  Easy |
| 4.9 | To rise from the lying or sitting position, my dog finds it:  Difficult  Easy |
| 4.10 | My dog indicates vocally if in pain when touched:  Rarely  Often |
| 4.11 | At the start of the day, my dog’s stiffness is:  Not noticeable  Severe |
| 4.12 | At the end of the day, my dog’s stiffness is:  Not noticeable  Severe |
| 4.13 | My dog shows lameness when walking:  Never  Frequently |
| 4.14 | My dog shows lameness when trotting:  Never  Frequently |
| 4.15 | My dog shows lameness when sprinting/galloping/cantering:  Never  Frequently |
| 4.16 | My dog indicates pain when turning:  Never  Frequently |
| 4.17 | My dog is a working dog: Y / N  If yes, where does your dog work:   |  | | --- | |
| 4.18 | My dog participates in dog shows:  Never  Frequently  *If your dog participates or participated in dog shows, please provide some further details:*  Please describe the show activities your dog participates/ participated in:   |  | | --- | |  | |  | |
|  | | How many days per month does/ did your dog participate? | to per month | | --- | --- | | How much training per month does/ did your dog receive? | to per month | | How long does/ did each training session last (in hours)? | to hours | | Since what age has your dog participated? | years, months | | If your dog no longer participates, what age did it retire? | years, months | | Do you warm your dog up before training/ competition? | Y / N / Sometimes | | If Yes, or Sometimes, please provide some details:   | Please select the method(s) of warm up:  Stretching Walking Playing Pre-planned warm up routine  Other (please specify): _______________________________ | | | --- | --- | | How long does each warm up last (minutes)? | to minutes | | | | Do you cool your dog down after training/ competition? | Y / N / Sometimes | | If Yes, or Sometimes, please provide some details:   | Please select the method(s) of cool down:  Stretching Walking Playing Pre-planned cool down routine  Other (please specify): _______________________________ | | | --- | --- | | How long does each cool down last (minutes)? | to minutes | | | |
| 4.19 | My dog practices sport, working trials or training:  Never  Frequently |
|  | *If your dog practices or practiced sports or working trials, please provide some further details:*  Please describe the sports or working trials your dog practices/ practiced in and its level in the sport:   |  | | --- | |  | |  | |
|  | | How many days per month does/ did your dog practice? | to per month | | --- | --- | | How frequently does your dog compete in the sport/ working trial?  per month, per year, Never | | | Since what age has your dog practiced? | years, months | | If your dog no longer practices, what age did it retire? | years, months | | Do you warm your dog up before training/ sport? | Y / N / Sometimes | | If Yes, or Sometimes, please provide some details:   | Please select the method(s) of warm up:  Stretching Walking Playing Pre-planned warm up routine  Other (please specify): _______________________________ | | | --- | --- | | How long does each warm up last (minutes)? | to minutes | | | | Do you cool your dog down after training/ sport? | Y / N / Sometimes | | If Yes, or Sometimes, please provide some details:   | Please select the method(s) of cool down:  Stretching Walking Playing Pre-planned cool down routine  Other (please specify): _______________________________ | | | --- | --- | | How long does each cool down last (minutes)? | to minutes | | | |
| 4.20 | | Has your dog been injured in the past FIVE years? | Y / N | | --- | --- | |
|  | *If Yes, please provide some further details of each injury your dog has had in the past FIVE years:* |
|  | | What age was your dog when the injury occurred (years, months)?   | Injury: | Injury 1 | Injury 2 | Injury 3 | Injury 4 | | --- | --- | --- | --- | --- | | Age: |  |  |  |  | | | | --- | --- | --- | --- | --- | --- | --- | --- | --- | --- | --- | --- | | Did the injury occur during training, competition or sport?   | Injury: | Injury 1 | Injury 2 | Injury 3 | Injury 4 | | --- | --- | --- | --- | --- | | During competition? | Y / N | Y / N | Y / N | Y / N | | | | If Yes, please describe the session or sport when your dog was injured:   | Injury 1: |  | | --- | --- | | Injury 2: |  | | Injury 3: |  | | Injury 4: |  | | | | Please describe how your dog was injured:   | Injury 1: |  | | --- | --- | | Injury 2: |  | | Injury 3: |  | | Injury 4: |  | | | |  |  | | What was the type of injury?   | Injury: | Injury 1 | Injury 2 | Injury 3 | Injury 4 | | --- | --- | --- | --- | --- | | Broken/ fracture bone |  |  |  |  | | Muscle strain/ pull |  |  |  |  | | Ligament damage |  |  |  |  | | Abrasion |  |  |  |  | | Cut |  |  |  |  | | Other |  |  |  |  |   *If other, please specify:* __________________________________________  Please describe where in the body your dog was injured:   | Injury 1: |  | | --- | --- | | Injury 2: |  | | Injury 3: |  | | Injury 4: |  |   Has this region been injured before?   | Injury: | Injury 1 | Injury 2 | Injury 3 | Injury 4 | | --- | --- | --- | --- | --- | | Region injured before? | Y / N /  Don’t know | Y / N /  Don’t know | Y / N /  Don’t know | Y / N /  Don’t know |   Has your dog made a full recovery from this injury?   | Injury: | Injury 1 | Injury 2 | Injury 3 | Injury 4 | | --- | --- | --- | --- | --- | | Fully recovered? | Y / N | Y / N | Y / N | Y / N | | | |
|  |  |
| 4.21 | | Would you be willing to participate in any future study with us? | Y / N | | --- | --- | |
|  |  |

**Thank you for your time in answering this questionnaire.**
