# Supplementary material for: Different conformations of the German shepherd dog breed affect its posture and movement
Source: Sci Rep. 2020 Oct 15;10:16924. doi: 10.1038/s41598-020-73550-x (PMC7567065; doi:10.1038/s41598-020-73550-x)
Supplement: Supplementary file 2 — Supplementary Information 2. [file 41598_2020_73550_MOESM2_ESM.docx]

Different conformations of the German shepherd dog breed affect its posture and movement

A Humphries^1^, AF Shaheen^1,2^, CB Gomez Alvarez^1, 2, 3*^

^1^ School of Veterinary Medicine, University of Surrey, Guildford, UK; ^2^ Department of Life Sciences, Brunel University London, UK; ^3^  Department of Veterinary Medicine, University of Cambridge, UK

* corresponding author= Constanza Gomez Alvarez [c.gomezalvarez@surrey.ac.uk](mailto:c.gomezalvarez@surrey.ac.uk)

Supplementary material:

**S2: Conformation**

Conformation parameters, signalment, fitness parameters and hip scores (mean and inter-subject standard deviations) for the breed and each back slope and curvature group. Chi-square tests are conducted for nominal variables and Kruskal-Wallis (KW) for non-nominal values, when p-values for these tests were significant (p<0.05), post hoc p-values are reported. The groups are abbreviated as follows: Le (Levelled), In (intermediate) and Sl (sloped). Significance is set at p<0.05 and significant values are highlighted.

| **Group:** | **Whole group** | | **Back slope** | | | | | |  | | | | **Back curvature** | | | | | |
| --- | --- | --- | --- | --- | --- | --- | --- | --- | --- | --- | --- | --- | --- | --- | --- | --- | --- | --- |
|  |  |  | **Levelled** | | **Intermediate** | | **Sloped** | | **Chi- Square p-value** | **post hoc p-value** | | | **Straight** | | | **Arched** | | **Chi- Square p-value** |
|  |  |  |  |  |  |  |  |  |  | **Le** | **In** | **Sl** |  |  |  |  |  |  |
| Sex (% Male) | 48.08 | | 33.33 | | 52.38 | | 61.90 | | 0.198 |  |  |  | 38.71 | | 62.07 | | | 0.071 |
| Straight/ arched back curvature (% Straight) | 51.67 | | 72.22 | | 52.38 | | 33.33 | | 0.053 |  |  |  | 10.00 | | 0.00 | | | 0.00 |
| Active in agility (% of the group) | 38.33 | | 38.89 | | 38.10 | | 38.10 | | 0.998 |  |  |  | 41.94 | | 34.48 | | | 0.553 |
| Participates in breed shows (% of the group) | 41.67 | | 16.67 | | 38.10 | | 66.67 | | 0.006 | 0.027 | 1.000 | 0.011 | 25.81 | | 58.62 | | | 0.010 |
|  | **Mean** | **SD** | **Mean** | **SD** | **Mean** | **SD** | **Mean** | **SD** | **KW**  **p-value** | **Dunn’s p-value** | | | **Mean** | **SD** | **Mean** | | **SD** | **KW p-value** |
|  |  |  |  |  |  |  |  |  |  | **Le-In** | **Le-Sl** | **In-Sl** |  |  |  |  |  |  |
| Age | 4.32 | 2.34 | 4.95 | 2.11 | 4.10 | 2.32 | 3.99 | 2.54 | 0.255 |  |  |  | 4.24 | 2.36 | 4.39 | | 2.35 | 0.767 |
| Height withers (cm) | 63.00 | 3.33 | 62.42 | 2.38 | 62.36 | 3.51 | 64.14 | 3.66 | 0.164 |  |  |  | 62.31 | 3.00 | 63.74 | | 3.55 | 0.098 |
| Height sacrum (cm) | 58.29 | 3.47 | 60.96 | 2.42 | 58.12 | 3.23 | 56.17 | 2.99 | <0.001 | 0.012 | <0.001 | 0.281 | 58.63 | 3.46 | 57.92 | | 3.50 | 0.463 |
| Length of back (cm) | 57.25 | 4.10 | 57.78 | 4.83 | 55.57 | 3.68 | 58.48 | 3.37 | 0.034 | 0.272 | 1.000 | 0.033 | 57.06 | 4.04 | 57.45 | | 4.22 | 0.593 |
| Slope of back (°) | 4.67 | 2.75 | 1.44 | 1.01 | 4.35 | 0.83 | 7.74 | 1.12 | <0.001 | 0.002 | <0.001 | <0.001 | 3.67 | 2.42 | 5.73 | | 2.71 | 0.003 |
| Thorax radius of curvature (R_T_) (m) | 2.26 | 4.00 | 4.49 | 6.57 | 1.28 | 0.88 | 1.21 | 1.58 | 0.022 | 0.574 | 0.017 | 0.418 | 3.76 | 5.09 | 0.57 | | 0.26 | <0.001 |
| Lumbar radius of curvature (R_L_) (m) | 0.40 | 0.07 | 0.40 | 0.05 | 0.41 | 0.09 | 0.39 | 0.06 | 0.975 |  |  |  | 0.38 | 0.05 | 0.41 | | 0.08 | 0.100 |
| Body Condition score (1-9) | 4.93 | 0.77 | 4.83 | 0.99 | 4.98 | 0.80 | 4.98 | 0.51 | 0.940 |  |  |  | 4.89 | 0.89 | 4.98 | | 0.62 | 0.889 |
| Right thigh circumference (cm) | 37.29 | 3.13 | 37.19 | 2.60 | 38.50 | 3.75 | 36.15 | 2.48 | 0.163 |  |  |  | 37.51 | 3.33 | 37.05 | | 2.93 | 0.864 |
| Left thigh circumference (cm) | 37.08 | 2.97 | 37.33 | 2.63 | 37.57 | 3.26 | 36.36 | 2.92 | 0.539 |  |  |  | 37.02 | 3.08 | 37.14 | | 2.89 | 0.593 |
| Muscle tone left side quadriceps (1-3) | 2.06 | 0.32 | 2.11 | 0.37 | 2.10 | 0.41 | 1.98 | 0.11 | 0.262 |  |  |  | 1.97 | 0.22 | 2.16 | | 0.38 | 0.042 |
| Muscle tone right side quadriceps (1-3) | 2.05 | 0.31 | 2.08 | 0.35 | 2.10 | 0.41 | 1.98 | 0.11 | 0.288 |  |  |  | 1.97 | 0.22 | 2.14 | | 0.38 | 0.076 |
| Muscle tone left side triceps (1-3) | 2.03 | 0.19 | 2.03 | 0.27 | 2.05 | 0.22 | 2.00 | 0.00 | 0.738 |  |  |  | 1.98 | 0.09 | 2.07 | | 0.26 | 0.081 |
| Muscle tone right side triceps (1-3) | 2.03 | 0.19 | 2.03 | 0.27 | 2.05 | 0.22 | 2.00 | 0.00 | 0.738 |  |  |  | 1.98 | 0.09 | 2.07 | | 0.26 | 0.081 |
| Fat thickness at right side of L4 (mm) | 8.04 | 2.56 | 7.92 | 3.63 | 8.52 | 1.97 | 7.67 | 1.96 | 0.485 |  |  |  | 7.74 | 2.41 | 8.36 | | 2.72 | 0.589 |
| Fat thickness at left side of L4 (mm) | 8.09 | 2.45 | 7.92 | 3.58 | 8.52 | 1.94 | 7.81 | 1.66 | 0.500 |  |  |  | 7.77 | 2.22 | 8.43 | | 2.68 | 0.585 |
| Hip score | 8.00 | 3.72 | 8.50 | 3.33 | 8.33 | 3.75 | 7.24 | 4.06 | 0.850 |  |  |  | 7.81 | 3.59 | 8.21 | | 3.91 | 0.508 |

**S3: Marker locations**

List of anatomical markers and clusters (clust) used to track the left side and spine of the GSDs. All markers were repeated for the right side, except the spine clusters.

| **Segment** | **Marker name** | **Anatomical landmark** |
| --- | --- | --- |
| **Pelvis:** | IW | Ilial wing |
|  | IT | Ischial tuberosity |
| **Femur:** | GT | Greater Trochanter |
|  | LSF | Lateral Femoral condyle (stifle) |
|  | Quad (clust) | Quadriceps (anterior-lateral surface). |
| **Tibia:** | LSF | Lateral Femoral condyle (stifle) |
|  | LHK | Lateral Malleolus (hock) |
|  | Gast (clust) | Gastrocnemius (junction between muscle and tendon) |
| **Metatarsals:** | LHK | Lateral Malleolus (hock) |
|  | MT | 5^th^ metatarsophalangeal joint |
|  | Metat (clust) | Metatarsals anterior surface |
| **Scapula:** | TS | Proximal scapula spine |
|  | AA | Acromion |
|  | AI | Caudal angle of the scapula |
|  | Scap (clust) | Scapula half way along scapula spine |
| **Humerus:** | HH | Greater Humeral Tubercle |
|  | LEB | Lateral Humeral Epicondyle (elbow) |
|  | Hum (clust) | Humerus anterior surface |
| **Radius:** | LEB | Lateral Epicondyle (elbow) |
|  | LCP | Lateral Styloid process (carpus) |
|  | Rad (clust) | Radius anterior surface |
| **Metacarpals:** | LCP | Lateral Styloid process (carpus) |
|  | MC | 5^th^ metacarpophalangeal joint |
|  | Metac (clust) | Metacarpals anterior surface |
| **Head:** | Head (clust) | Skull, half way along sagittal crest |
| **Back:** | With (clust) | Withers (highest point on back) |
|  | T8 (clust) | Thorax, 8th thoracic vertebrae |
|  | L1 (clust) | Lumbar, 1st lumbar vertebrae |
|  | L5 (clust) | Lumbar, 5th Lumbar vertebrae |
|  | Sacr (clust) | Sacrum, Median crest |
| **Tail:** | Co3 (clust) | Proximal tail, 3rd Coccygeal vertebrae |
|  | Co8 (clust) | Middle tail, 8th Coccygeal vertebrae |
|  | Co14 (clust) | Distal tail, 14th Coccygeal vertebrae |

**S4: Postural kinetics**

Kinetic parameters normalised to body weight, recorded when standing square (mean and inter-subject standard deviations) for the breed and each slope group. Normality p-values show if parameters are normally distributed (p>0.05). The correlation coefficient (r) and p-values show the correlation between the parameter and the slope of the back (significant correlation highlighted (p<0.05)). When Kruskal-Wallis (KW) p-values were significant, Dunn posthoc values were calculated between the groups: levelled (Le), intermediate (In) and sloped (Sl). (FL: forelimb, HL: hind limb, BW: body weight, met: metacarpal/ metatarsal pad, dig: digital pads).

| **Kinetic parameter** | **Whole group** | | **Normality p-value** | **Correlation with slope** | | **Levelled** | | **Intermediate** | | **Sloped** | | **KW**  **p-value** | **Dunn posthoc** | | |
| --- | --- | --- | --- | --- | --- | --- | --- | --- | --- | --- | --- | --- | --- | --- | --- |
|  | **Mean** | **SD** |  | **r** | **p-value** | **Mean** | **SD** | **Mean** | **SD** | **Mean** | **SD** |  | **Le-In** | **Le-Sl** | **In-Sl** |
| Right FL % Weight bearing | 49.19 | 3.76 | 0.228 | 0.16 | 0.218 | 48.32 | 4.46 | 49.10 | 3.60 | 49.98 | 3.30 | 0.339 | n/a | n/a | n/a |
| Left FL % Weight bearing | 50.81 | 3.76 | 0.228 | -0.16 | 0.218 | 51.68 | 4.46 | 50.90 | 3.60 | 50.02 | 3.30 | 0.339 | n/a | n/a | n/a |
| Right HL % Weight bearing | 50.42 | 5.37 | 0.132 | -0.01 | 0.928 | 50.62 | 4.01 | 50.44 | 5.51 | 50.23 | 6.45 | 0.937 | n/a | n/a | n/a |
| Left HL % Weight bearing | 49.58 | 5.37 | 0.132 | 0.01 | 0.928 | 49.38 | 4.01 | 49.56 | 5.51 | 49.77 | 6.45 | 0.937 | n/a | n/a | n/a |
| FL % Weight bearing | 62.16 | 2.94 | 0.561 | 0.26 | 0.042 | 60.75 | 3.68 | 63.21 | 2.25 | 62.26 | 2.50 | 0.076 | n/a | n/a | n/a |
| HL % Weight bearing | 37.84 | 2.94 | 0.561 | -0.26 | 0.042 | 39.25 | 3.68 | 36.79 | 2.25 | 37.74 | 2.50 | 0.076 | n/a | n/a | n/a |
| **Right Forelimb** | | | | | | | | | | | | | | | |
|  |  |  |  |  |  |  |  |  |  |  |  |  |  |  |  |
| Vertical force (%BW) | 30.19 | 2.81 | 0.439 | 0.18 | 0.177 | 28.91 | 2.97 | 30.94 | 2.07 | 30.51 | 3.11 | 0.064 | n/a | n/a | n/a |
| Contact area (cm^2^/kg) | 0.45 | 0.07 | 0.445 | 0.44 | <0.001 | 0.41 | 0.05 | 0.46 | 0.09 | 0.49 | 0.05 | 0.001 | 0.087 | <0.001 | 0.236 |
| Peak vertical force (%BW) | 3.97 | 0.84 | 0.709 | -0.31 | 0.016 | 4.09 | 0.81 | 4.21 | 0.75 | 3.63 | 0.87 | 0.070 | n/a | n/a | n/a |
| Vertical force_dig (%BW) | 19.48 | 4.84 | 0.071 | -0.17 | 0.193 | 19.34 | 4.29 | 21.04 | 3.97 | 18.11 | 5.73 | 0.159 | n/a | n/a | n/a |
| Vertical force_met (%BW) | 10.18 | 4.35 | 0.335 | 0.25 | 0.050 | 9.18 | 4.62 | 9.48 | 4.19 | 11.82 | 3.97 | 0.104 | n/a | n/a | n/a |
| Contact area_dig (cm^2^/kg) | 0.26 | 0.04 | 0.407 | 0.19 | 0.151 | 0.25 | 0.04 | 0.27 | 0.04 | 0.27 | 0.04 | 0.063 | n/a | n/a | n/a |
| Contact area_met (cm^2^/kg) | 0.19 | 0.05 | 0.914 | 0.44 | <0.001 | 0.17 | 0.03 | 0.18 | 0.06 | 0.22 | 0.05 | 0.006 | 0.788 | 0.005 | 0.115 |
| Peak vertical force_dig (%BW) | 3.87 | 0.96 | 0.200 | -0.31 | 0.014 | 4.03 | 0.93 | 4.18 | 0.81 | 3.43 | 1.01 | 0.029 | 0.147 | 1.000 | 0.035 |
| Peak vertical force_met (%BW) | 2.09 | 0.6 | 0.672 | 0.03 | 0.835 | 2.08 | 0.71 | 2.02 | 0.64 | 2.17 | 0.47 | 0.588 | n/a | n/a | n/a |
| **Left Forelimb** | | | | | | | | | | | | | | | |
| Vertical force (%BW) | 31.12 | 3.21 | 0.146 | -0.11 | 0.393 | 31.22 | 3.49 | 31.71 | 2.35 | 30.48 | 3.69 | 0.587 | n/a | n/a | n/a |
| Contact area (cm^2^/kg) | 0.46 | 0.07 | 0.243 | 0.37 | 0.003 | 0.42 | 0.06 | 0.47 | 0.08 | 0.49 | 0.06 | 0.007 | 0.081 | 0.006 | 1.000 |
| Peak vertical force (%BW) | 4.00 | 0.76 | 0.165 | -0.16 | 0.222 | 4.02 | 0.65 | 4.14 | 0.67 | 3.83 | 0.93 | 0.315 | n/a | n/a | n/a |
| Vertical force_dig (%BW) | 18.78 | 4.72 | 0.315 | -0.04 | 0.737 | 17.49 | 4.47 | 20.74 | 4.18 | 17.69 | 4.96 | 0.037 | 0.101 | 1.000 | 0.075 |
| Vertical force_met (%BW) | 12.19 | 4.76 | 0.43 | -0.10 | 0.431 | 13.37 | 4.53 | 11.38 | 4.83 | 12.15 | 4.90 | 0.344 | n/a | n/a | n/a |
| Contact area_dig (cm^2^/kg) | 0.26 | 0.04 | 0.225 | 0.34 | 0.008 | 0.23 | 0.04 | 0.27 | 0.04 | 0.27 | 0.04 | 0.005 | 0.016 | 0.010 | 1.000 |
| Contact area_met (cm^2^/kg) | 0.20 | 0.06 | 0.505 | 0.29 | 0.023 | 0.19 | 0.04 | 0.20 | 0.06 | 0.22 | 0.06 | 0.131 | n/a | n/a | n/a |
| Peak vertical force_dig(%BW) | 3.85 | 0.92 | 0.360 | -0.17 | 0.184 | 3.90 | 0.82 | 4.06 | 0.82 | 3.60 | 1.08 | 0.188 | n/a | n/a | n/a |
| Peak vertical force_met (%BW) | 2.30 | 0.65 | 0.219 | -0.18 | 0.168 | 2.45 | 0.65 | 2.24 | 0.59 | 2.22 | 0.71 | 0.445 | n/a | n/a | n/a |
| **Right Hind limb** | | | | | | | | | | | | | | | |
| Vertical force (%BW) | 18.98 | 2.49 | 0.414 | -0.27 | 0.036 | 19.99 | 2.27 | 18.49 | 2.20 | 18.66 | 2.83 | 0.113 | n/a | n/a | n/a |
| Contact area (cm^2^/kg) | 0.31 | 0.05 | 0.558 | 0.05 | 0.694 | 0.31 | 0.05 | 0.31 | 0.06 | 0.32 | 0.05 | 0.600 | n/a | n/a | n/a |
| Peak vertical force (%BW) | 3.39 | 0.54 | 0.177 | -0.17 | 0.207 | 3.37 | 0.42 | 3.48 | 0.59 | 3.32 | 0.60 | 0.670 | n/a | n/a | n/a |
| Vertical force_dig (%BW) | 15.60 | 2.47 | 0.233 | -0.09 | 0.509 | 15.50 | 2.00 | 15.89 | 2.52 | 15.36 | 2.85 | 0.810 | n/a | n/a | n/a |
| Vertical force_met (%BW) | 2.90 | 1.62 | 0.176 | -0.39 | 0.002 | 3.83 | 1.71 | 2.61 | 1.40 | 2.46 | 1.54 | 0.034 | 0.086 | 0.049 | 1.000 |
| Contact area_dig (cm^2^/kg) | 0.24 | 0.05 | 0.578 | 0.23 | 0.073 | 0.23 | 0.03 | 0.24 | 0.05 | 0.25 | 0.05 | 0.087 | n/a | n/a | n/a |
| Contact area_met (cm^2^/kg) | 0.07 | 0.03 | 0.310 | -0.28 | 0.029 | 0.08 | 0.02 | 0.06 | 0.03 | 0.06 | 0.03 | 0.046 | 0.075 | 0.107 | 1.000 |
| Peak vertical force_dig (%BW) | 3.35 | 0.55 | 0.109 | -0.08 | 0.531 | 3.27 | 0.41 | 3.47 | 0.59 | 3.29 | 0.62 | 0.787 | n/a | n/a | n/a |
| Peak vertical force_met (%BW) | 1.31 | 0.64 | 0.273 | -0.36 | 0.005 | 1.67 | 0.68 | 1.17 | 0.54 | 1.14 | 0.6 | 0.035 | 0.086 | 0.056 | 1.000 |
| **Left Hind limb** | | | | | | | | | | | | | | | |
| Vertical force (%BW) | 18.25 | 3.16 | 0.005 | -0.24 | 0.067 | 18.74 | 3.47 | 18.19 | 2.45 | 17.87 | 3.60 | 0.657 | n/a | n/a | n/a |
| Contact area (cm^2^/kg) | 0.30 | 0.05 | 0.438 | -0.04 | 0.790 | 0.30 | 0.05 | 0.30 | 0.05 | 0.31 | 0.06 | 0.769 | n/a | n/a | n/a |
| Peak vertical force (%BW) | 3.31 | 0.57 | 0.490 | -0.02 | 0.864 | 3.20 | 0.39 | 3.38 | 0.55 | 3.33 | 0.72 | 0.673 | n/a | n/a | n/a |
| Vertical force_dig (%BW) | 15.43 | 2.60 | 0.030 | -0.19 | 0.153 | 15.66 | 2.45 | 15.62 | 2.30 | 15.04 | 3.06 | 0.837 | n/a | n/a | n/a |
| Vertical force_met (%BW) | 2.95 | 1.93 | 0.074 | -0.16 | 0.233 | 3.47 | 1.71 | 2.32 | 1.75 | 3.17 | 2.21 | 0.118 | n/a | n/a | n/a |
| Contact area_dig (cm^2^/kg) | 0.24 | 0.03 | 0.014 | 0.14 | 0.292 | 0.23 | 0.02 | 0.25 | 0.04 | 0.25 | 0.03 | 0.095 | n/a | n/a | n/a |
| Contact area_met (cm^2^/kg) | 0.07 | 0.03 | 0.274 | -0.03 | 0.806 | 0.07 | 0.02 | 0.05 | 0.03 | 0.08 | 0.04 | 0.091 | n/a | n/a | n/a |
| Peak vertical force_dig(%BW) | 3.34 | 0.58 | 0.112 | 0.06 | 0.662 | 3.19 | 0.41 | 3.36 | 0.57 | 3.45 | 0.72 | 0.509 | n/a | n/a | n/a |
| Peak vertical force_met (%BW) | 1.19 | 0.66 | 0.335 | -0.20 | 0.120 | 1.42 | 0.60 | 0.95 | 0.59 | 1.24 | 0.74 | 0.053 | n/a | n/a | n/a |

**(Continued S4): Postural kinetics**

Kinetic parameters normalised to body weight, recorded in the stacked position (mean and inter-subject standard deviations) for the breed and each slope group. Normality p-values show if parameters are normally distributed (p>0.05). The correlation coefficient (r) and p-values show the correlation between the parameter and the slope of the back (significant correlation highlighted (p<0.05)). When Kruskal-Wallis (KW) p-values were significant, Dunn posthoc values were calculated between the groups: levelled (Le), intermediate (In) and sloped (Sl). (FL: forelimb, HL: hind limb, BW: body weight, met: metacarpal/ metatarsal pad, dig: digital pads).

| **Kinetic parameter** | **Whole group** | | | **Normality p-value** | | **Correlation with slope** | | **Levelled** | | **Intermediate** | | **Sloped** | | **KW**  **p-value** | **Dunn posthoc** | | |
| --- | --- | --- | --- | --- | --- | --- | --- | --- | --- | --- | --- | --- | --- | --- | --- | --- | --- |
|  | **Mean** | **SD** | |  |  | **r** | **p-value** | **Mean** | **SD** | **Mean** | **SD** | **Mean** | **SD** |  | **Le-In** | **Le-Sl** | **In-Sl** |
| Right FL % Weight bearing | 49.78 | 2.76 | | 0.078 | | -0.05 | 0.698 | 49.49 | 3.50 | 50.05 | 2.93 | 49.75 | 1.93 | 0.783 | n/a | n/a | n/a |
| Left FL % Weight bearing | 50.22 | 2.76 | | 0.078 | | 0.05 | 0.698 | 50.51 | 3.50 | 49.95 | 2.93 | 50.25 | 1.93 | 0.783 | n/a | n/a | n/a |
| Right HL % Weight bearing | 48.61 | 10.81 | | 0.112 | | -0.18 | 0.171 | 53.03 | 6.21 | 45.27 | 12.34 | 47.77 | 11.69 | 0.094 | n/a | n/a | n/a |
| Left HL % Weight bearing | 51.39 | 10.81 | | 0.112 | | 0.18 | 0.171 | 46.97 | 6.21 | 54.73 | 12.34 | 52.23 | 11.69 | 0.094 | n/a | n/a | n/a |
| FL % Weight bearing | 61.70 | 2.63 | | 0.183 | | 0.33 | 0.010 | 60.52 | 2.89 | 61.98 | 2.42 | 62.51 | 2.31 | 0.034 | 0.218 | 0.011 | 1.000 |
| HL % Weight bearing | 38.30 | 2.63 | | 0.183 | | -0.33 | 0.010 | 39.48 | 2.89 | 38.02 | 2.42 | 37.49 | 2.31 | 0.034 | 0.218 | 0.033 | 1.000 |
| **Right Forelimb** | | | | | | | | | | | | | | | | | |
| Vertical force (%BW) | 30.14 | 3.18 | | 0.064 | | 0.33 | 0.010 | 28.31 | 2.00 | 30.73 | 3.26 | 31.04 | 3.38 | 0.012 | 0.029 | 0.025 | 1.000 |
| Contact area (cm^2^/kg) | 0.47 | 0.07 | | 0.024 | | 0.45 | <0.001 | 0.42 | 0.05 | 0.47 | 0.07 | 0.50 | 0.07 | 0.003 | 0.150 | 0.002 | 0.404 |
| Peak vertical force (%BW) | 3.78 | 0.74 | | 0.762 | | -0.08 | 0.528 | 3.77 | 0.5 | 4.00 | 0.86 | 3.60 | 0.81 | 0.368 | n/a | n/a | n/a |
| Vertical force_dig (%BW) | 17.27 | 4.53 | | 0.364 | | 0.10 | 0.442 | 16.35 | 2.84 | 18.31 | 4.51 | 17.06 | 5.56 | 0.137 | n/a | n/a | n/a |
| Vertical force_met (%BW) | 12.54 | 4.04 | | 0.689 | | 0.20 | 0.133 | 12.09 | 2.97 | 11.26 | 4.03 | 13.99 | 4.49 | 0.128 | n/a | n/a | n/a |
| Contact area_dig (cm^2^/kg) | 0.26 | 0.04 | | 0.112 | | 0.19 | 0.135 | 0.25 | 0.05 | 0.27 | 0.04 | 0.27 | 0.04 | 0.034 | 1.000 | 0.038 | 0.186 |
| Contact area_met (cm^2^/kg) | 0.21 | 0.05 | | 0.094 | | 0.33 | 0.011 | 0.19 | 0.05 | 0.20 | 0.05 | 0.24 | 0.05 | 0.213 | n/a | n/a | n/a |
| Peak vertical force_dig (%BW) | 3.63 | 0.85 | | 0.243 | | -0.08 | 0.551 | 3.64 | 0.62 | 3.89 | 0.89 | 3.39 | 0.95 | 0.435 | n/a | n/a | n/a |
| Peak vertical force_met (%BW) | 2.35 | 0.49 | | 0.139 | | -0.06 | 0.656 | 2.42 | 0.56 | 2.27 | 0.44 | 2.37 | 0.49 | 0.073 | n/a | n/a | n/a |
| **Left Forelimb** | | | | | | | | | | | | | | | | | |
| Vertical force (%BW) | 30.14 | | 3.74 | | 0.607 | 0.40 | 0.001 | 28.53 | 3.94 | 30.08 | 3.70 | 31.57 | 3.15 | 0.073 | n/a | n/a | n/a |
| Contact area (cm^2^/kg) | 0.48 | | 0.08 | | 0.170 | 0.44 | <0.001 | 0.44 | 0.07 | 0.47 | 0.08 | 0.52 | 0.07 | 0.003 | 0.387 | 0.002 | 0.168 |
| Peak vertical force (%BW) | 3.59 | | 0.61 | | 0.277 | 0.04 | 0.767 | 3.58 | 0.59 | 3.64 | 0.64 | 3.56 | 0.63 | 0.922 | n/a | n/a | n/a |
| Vertical force_dig (%BW) | 16.55 | | 4.35 | | 0.174 | 0.22 | 0.088 | 15.64 | 4.04 | 16.73 | 3.90 | 17.16 | 5.01 | 0.520 | n/a | n/a | n/a |
| Vertical force_met (%BW) | 13.62 | | 4.26 | | 0.234 | 0.12 | 0.348 | 13.19 | 4.16 | 13.35 | 4.10 | 14.24 | 4.64 | 0.630 | n/a | n/a | n/a |
| Contact area_dig (cm^2^/kg) | 0.25 | | 0.04 | | 0.475 | 0.35 | 0.007 | 0.23 | 0.04 | 0.25 | 0.05 | 0.27 | 0.04 | 0.032 | 0.209 | 0.032 | 1.000 |
| Contact area_met (cm^2^/kg) | 0.23 | | 0.05 | | 0.003 | 0.43 | 0.001 | 0.21 | 0.05 | 0.23 | 0.05 | 0.26 | 0.06 | 0.012 | 0.467 | 0.009 | 0.399 |
| Peak vertical force_dig (%BW) | 3.37 | | 0.74 | | 0.610 | 0.13 | 0.332 | 3.20 | 0.73 | 3.58 | 0.61 | 3.33 | 0.83 | 0.346 | n/a | n/a | n/a |
| Peak vertical force_met (%BW) | 2.55 | | 0.57 | | 0.522 | -0.06 | 0.642 | 2.59 | 0.57 | 2.53 | 0.52 | 2.53 | 0.63 | 0.902 | n/a | n/a | n/a |
| **Right Hind limb** | | | | | | | | | | | | | | | | | |
| Vertical force (%BW) | 18.13 | | 5.61 | | 0.441 | -0.09 | 0.493 | 19.70 | 4.17 | 17.06 | 6.45 | 17.82 | 5.78 | 0.215 | n/a | n/a | n/a |
| Contact area (cm^2^/kg) | 0.33 | | 0.07 | | 0.707 | 0.14 | 0.280 | 0.34 | 0.06 | 0.31 | 0.08 | 0.35 | 0.07 | 0.298 | n/a | n/a | n/a |
| Peak vertical force (%BW) | 2.96 | | 0.72 | | 0.624 | -0.09 | 0.475 | 3.12 | 0.66 | 2.88 | 0.79 | 2.91 | 0.71 | 0.564 | n/a | n/a | n/a |
| Vertical force_dig (%BW) | 11.45 | | 4.33 | | 0.146 | -0.09 | 0.504 | 12.41 | 4.24 | 10.99 | 4.07 | 11.04 | 4.67 | 0.550 | n/a | n/a | n/a |
| Vertical force_met (%BW) | 6.64 | | 2.64 | | 0.571 | -0.08 | 0.552 | 7.45 | 1.76 | 5.81 | 2.50 | 6.78 | 3.16 | 0.149 | n/a | n/a | n/a |
| Contact area_dig (cm^2^/kg) | 0.22 | | 0.05 | | 0.353 | 0.07 | 0.575 | 0.22 | 0.04 | 0.20 | 0.05 | 0.22 | 0.05 | 0.499 | n/a | n/a | n/a |
| Contact area_met (cm^2^/kg) | 0.12 | | 0.04 | | 0.191 | 0.15 | 0.249 | 0.11 | 0.02 | 0.11 | 0.04 | 0.13 | 0.05 | 0.359 | n/a | n/a | n/a |
| Peak vertical force_dig (%BW) | 2.60 | | 0.80 | | 0.201 | 0.02 | 0.898 | 2.59 | 0.79 | 2.69 | 0.88 | 2.53 | 0.77 | 0.890 | n/a | n/a | n/a |
| Peak vertical force_met (%BW) | 2.33 | | 0.82 | | 0.072 | -0.12 | 0.376 | 2.55 | 0.69 | 2.19 | 0.86 | 2.27 | 0.89 | 0.380 | n/a | n/a | n/a |
| **Left Hind limb** | | | | | | | | | | | | | | | | | |
| Vertical force (%BW) | 18.85 | | 3.41 | | 0.255 | 0.28 | 0.028 | 17.63 | 1.85 | 18.92 | 3.00 | 19.90 | 4.45 | 0.084 | n/a | n/a | n/a |
| Contact area (cm^2^/kg) | 0.27 | | 0.05 | | 0.070 | 0.25 | 0.057 | 0.26 | 0.04 | 0.27 | 0.05 | 0.28 | 0.06 | 0.374 | n/a | n/a | n/a |
| Peak vertical force (%BW) | 4.28 | | 0.94 | | 0.097 | 0.12 | 0.345 | 3.89 | 0.53 | 4.50 | 0.89 | 4.41 | 1.15 | 0.088 | n/a | n/a | n/a |
| Vertical force_dig (%BW) | 18.40 | | 3.14 | | 0.468 | 0.26 | 0.047 | 17.04 | 1.69 | 18.92 | 2.85 | 19.10 | 4.00 | 0.047 | 0.139 | 0.066 | 1.000 |
| Vertical force_met (%BW) | 0.53 | | 0.94 | | <0.001 | -0.01 | 0.952 | 0.59 | 1.14 | 0.50 | 0.95 | 0.52 | 0.78 | 0.771 | n/a | n/a | n/a |
| Contact area_dig (cm^2^/kg) | 0.25 | | 0.05 | | 0.130 | 0.34 | 0.008 | 0.23 | 0.03 | 0.25 | 0.04 | 0.27 | 0.05 | 0.110 | n/a | n/a | n/a |
| Contact area_met (cm^2^/kg) | 0.01 | | 0.02 | | <0.001 | 0.10 | 0.464 | 0.01 | 0.03 | 0.01 | 0.02 | 0.02 | 0.02 | 0.702 | n/a | n/a | n/a |
| Peak vertical force_dig (%BW) | 4.36 | | 0.91 | | 0.093 | 0.15 | 0.251 | 3.91 | 0.54 | 4.57 | 0.88 | 4.51 | 1.08 | 0.060 | n/a | n/a | n/a |
| Peak vertical force_met (%BW) | 0.25 | | 0.42 | | <0.001 | 0.04 | 0.783 | 0.25 | 0.44 | 0.23 | 0.46 | 0.27 | 0.37 | 0.685 | n/a | n/a | n/a |

**S5: Postural Kinematics**

Kinematic flexion/ extension joint and segment angles recorded when standing square and in the stacked position (mean and inter-subject standard deviations) for the breed and each slope group. Normality p-values show if parameters are normally distributed (p>0.05). The correlation coefficient (r) and p-values show the correlation between the parameter and slope of the back (significant correlation highlighted (p<0.05)). When Kruskal-Wallis (KW) p-values were significant, Dunn’s posthoc values were calculated between the groups: levelled (Le), intermediate (In) and sloped (Sl). The anatomical markers used to calculate each joint angle in the Sagittal plane are shown in brackets.

| **Flexion (positive) and extension (negative) angle** | **Whole group** | | **Normality p-value** | **Correlation with slope** | | **Levelled** | | **Intermediate** | | **Sloped** | | **KW**  **p-value** | **Dunn’s posthoc** | | |
| --- | --- | --- | --- | --- | --- | --- | --- | --- | --- | --- | --- | --- | --- | --- | --- |
|  | **Mean** | **SD** |  | **r** | **p-value** | **Mean** | **SD** | **Mean** | **SD** | **Mean** | **SD** |  | **Le-In** | **Le-Sl** | **In-Sl** |
| **Standing square** | | | | | | | | | | | | | | | |
| Neck (Head-with-T8) | -15.88 | 10.60 | 0.744 | -0.04 | 0.782 | -19.03 | 12.22 | -12.43 | 8.23 | -16.82 | 10.95 | 0.227 | n/a | n/a | n/a |
| Mid Thorax (With-T8-L1) | -3.75 | 7.99 | 0.583 | 0.00 | 0.994 | -3.85 | 9.47 | -4.31 | 8.91 | -3.14 | 6.13 | 0.719 | n/a | n/a | n/a |
| Thoraco-lumbar (T8-L1-L5) | 2.36 | 7.46 | 0.504 | 0.43 | 0.001 | -0.37 | 5.50 | -0.21 | 8.17 | 6.63 | 6.32 | 0.002 | 1.000 | 0.006 | 0.016 |
| Lumbo-sacral (L1-L5-Sacr) | 11.05 | 4.55 | 0.735 | -0.01 | 0.936 | 11.15 | 3.74 | 10.70 | 4.64 | 11.28 | 5.12 | 0.947 | n/a | n/a | n/a |
| Tail set (L5-Sacr-Co3) | 35.05 | 6.36 | 0.179 | -0.26 | 0.044 | 37.80 | 5.90 | 33.35 | 7.05 | 34.11 | 5.53 | 0.056 | n/a | n/a | n/a |
| Co3 (Sacr-Co3-Co8) | 39.20 | 5.68 | 0.947 | -0.20 | 0.120 | 40.01 | 5.31 | 39.80 | 4.13 | 37.84 | 7.18 | 0.454 | n/a | n/a | n/a |
| Co8 (Co3-Co8-Co14) | 0.77 | 11.26 | 0.079 | 0.01 | 0.920 | 3.32 | 11.84 | -4.14 | 11.35 | 2.05 | 10.12 | 0.224 | n/a | n/a | n/a |
| Left Hip (IW-GT-LSF) | 29.67 | 10.78 | 0.635 | -0.41 | 0.001 | 36.57 | 9.74 | 28.05 | 9.39 | 25.88 | 10.58 | 0.019 | 0.069 | 0.025 | 1.000 |
| Right Hip (IW-GT-LSF) | 33.89 | 11.97 | 0.221 | -0.35 | 0.006 | 38.78 | 11.20 | 36.57 | 12.68 | 27.27 | 9.12 | 0.018 | 1.000 | 0.026 | 0.087 |
| Left Stifle (GT-LSF-LHK) | 44.07 | 8.44 | 0.092 | 0.02 | 0.861 | 45.75 | 6.57 | 41.75 | 8.75 | 44.69 | 9.45 | 0.297 | n/a | n/a | n/a |
| Right Stifle (GT-LSF-LHK) | 44.03 | 8.60 | 0.005 | 0.15 | 0.257 | 43.09 | 8.60 | 42.93 | 9.70 | 45.97 | 7.53 | 0.531 | n/a | n/a | n/a |
| Left Hock (LSF-LHK-MT) | 56.34 | 10.25 | 0.692 | 0.36 | 0.005 | 53.71 | 9.69 | 53.73 | 8.42 | 60.94 | 11.06 | 0.050 | n/a | n/a | n/a |
| Right Hock (LSF-LHK-MT) | 56.58 | 9.16 | 0.419 | 0.23 | 0.079 | 54.54 | 10.14 | 54.40 | 8.22 | 60.79 | 8.07 | 0.039 | 1.000 | 0.130 | 0.056 |
| Left Shoulder (TS-AA-LEB) | 65.01 | 10.06 | 0.073 | -0.24 | 0.079 | 70.15 | 5.20 | 63.30 | 10.63 | 60.72 | 12.16 | 0.108 | n/a | n/a | n/a |
| Right Shoulder (TS-AA-LEB) | 62.48 | 9.15 | 0.152 | -0.12 | 0.378 | 61.55 | 9.43 | 70.15 | 4.27 | 56.40 | 7.23 | 0.001 | 0.065 | 0.281 | <0.001 |
| Left Elbow (HH-LEB-LCP) | 42.92 | 7.00 | 0.754 | -0.04 | 0.777 | 42.81 | 7.55 | 42.97 | 6.36 | 43.01 | 7.53 | 0.967 | n/a | n/a | n/a |
| Right Elbow (HH-LEB-LCP) | 39.41 | 7.75 | 0.362 | 0.09 | 0.512 | 39.00 | 6.15 | 38.96 | 6.57 | 40.17 | 10.06 | 0.803 | n/a | n/a | n/a |
| Left Carpal (LEB-LCP-MC) | 34.67 | 8.92 | 0.061 | 0.41 | 0.001 | 29.91 | 8.53 | 34.88 | 8.65 | 38.48 | 7.96 | 0.014 | 0.279 | 0.011 | 0.592 |
| Right Carpal (LEB-LCP-MC) | 33.01 | 9.21 | 0.459 | -0.01 | 0.929 | 32.55 | 7.47 | 34.52 | 7.29 | 31.69 | 12.55 | 0.629 | n/a | n/a | n/a |

**(Continued S5): Postural kinematics**

| **Flexion (positive) and extension (negative) angle** | **Whole group** | | **Normality p-value** | **Correlation with slope** | | **Levelled** | | **Intermediate** | | **Sloped** | | **KW**  **p-value** | **Dunn posthoc** | | |
| --- | --- | --- | --- | --- | --- | --- | --- | --- | --- | --- | --- | --- | --- | --- | --- |
|  | **Mean** | **SD** |  | **r** | **p-value** | **Mean** | **SD** | **Mean** | **SD** | **Mean** | **SD** |  | **Le-In** | **Le-Sl** | **In-Sl** |
| **Stacked position** | | | | | | | | | | | | | | | |
| Neck (Head-with-T8) | -17.19 | 10.20 | 0.458 | -0.08 | 0.537 | -19.84 | 10.70 | -12.07 | 7.85 | -20.36 | 10.41 | 0.015 | 0.042 | 1.000 | 0.036 |
| Mid Thorax (With-T8-L1) | -3.78 | 5.98 | 0.095 | 0.03 | 0.791 | -2.98 | 4.84 | -6.25 | 5.81 | -1.98 | 6.30 | 0.059 | n/a | n/a | n/a |
| Thoraco-lumbar (T8-L1-L5) | 2.64 | 7.34 | 0.095 | 0.34 | 0.007 | 1.13 | 6.55 | -0.54 | 8.09 | 6.08 | 6.20 | 0.012 | 1.000 | 0.099 | 0.017 |
| Lumbo-sacral (L1-L5-Sacr) | 10.59 | 4.03 | 0.917 | 0.21 | 0.108 | 9.40 | 3.71 | 10.50 | 4.34 | 11.54 | 3.95 | 0.457 | n/a | n/a | n/a |
| Tail set (L5-Sacr-Co3) | 34.42 | 6.08 | 0.420 | -0.34 | 0.008 | 37.64 | 6.43 | 31.91 | 6.57 | 33.19 | 4.01 | 0.034 | 0.042 | 0.161 | 1.000 |
| Co3 (Sacr-Co3-Co8) | 38.05 | 6.44 | 0.633 | 0.05 | 0.692 | 37.10 | 6.99 | 39.46 | 3.53 | 38.10 | 7.48 | 0.356 | n/a | n/a | n/a |
| Co8 (Co3-Co8-Co14) | 5.09 | 8.30 | 0.350 | -0.25 | 0.055 | 9.46 | 8.33 | 0.73 | 4.52 | 4.80 | 8.97 | 0.045 | 0.039 | 0.406 | 0.701 |
| Left Hip (IW-GT-LSF) | 22.14 | 10.83 | 0.069 | -0.28 | 0.030 | 25.91 | 13.09 | 22.47 | 9.24 | 19.06 | 9.48 | 0.217 | n/a | n/a | n/a |
| Right Hip (IW-GT-LSF) | 39.55 | 12.32 | 0.409 | -0.18 | 0.174 | 41.59 | 10.92 | 44.92 | 14.04 | 33.36 | 9.92 | 0.030 | 1.000 | 0.159 | 0.038 |
| Left Stifle (GT-LSF-LHK) | 49.13 | 8.19 | 0.012 | 0.16 | 0.212 | 48.43 | 7.72 | 47.39 | 6.12 | 50.71 | 9.61 | 0.625 | n/a | n/a | n/a |
| Right Stifle (GT-LSF-LHK) | 42.80 | 8.07 | 0.084 | 0.22 | 0.091 | 39.70 | 8.03 | 46.31 | 6.70 | 43.13 | 8.36 | 0.125 | n/a | n/a | n/a |
| Left Hock (LSF-LHK-MT) | 54.42 | 9.32 | 0.440 | 0.15 | 0.256 | 54.02 | 12.63 | 52.61 | 5.65 | 56.34 | 9.20 | 0.343 | n/a | n/a | n/a |
| Right Hock (LSF-LHK-MT) | 63.39 | 9.66 | 0.479 | 0.29 | 0.023 | 60.30 | 6.46 | 61.53 | 9.59 | 67.62 | 10.82 | 0.045 | 1.000 | 0.060 | 0.184 |
| Left Shoulder (TS-AA-LEB) | 63.19 | 14.59 | 0.157 | -0.03 | 0.796 | 64.98 | 13.75 | 65.17 | 12.30 | 59.44 | 18.11 | 0.711 | n/a | n/a | n/a |
| Right Shoulder (TS-AA-LEB) | 62.62 | 9.23 | 0.272 | -0.10 | 0.448 | 63.22 | 10.92 | 66.92 | 8.65 | 58.38 | 5.95 | 0.021 | 0.789 | 0.243 | 0.019 |
| Left Elbow (HH-LEB-LCP) | 42.10 | 6.82 | 0.221 | 0.05 | 0.721 | 41.80 | 6.76 | 43.26 | 4.90 | 41.12 | 8.87 | 0.955 | n/a | n/a | n/a |
| Right Elbow (HH-LEB-LCP) | 37.80 | 6.30 | 0.813 | 0.13 | 0.313 | 37.28 | 5.19 | 37.46 | 6.87 | 38.61 | 7.03 | 0.785 | n/a | n/a | n/a |
| Left Carpal (LEB-LCP-MC) | 34.48 | 9.14 | 0.076 | 0.30 | 0.020 | 31.03 | 10.09 | 34.16 | 9.84 | 37.81 | 6.58 | 0.088 | n/a | n/a | n/a |
| Right Carpal (LEB-LCP-MC) | 33.51 | 8.82 | 0.090 | 0.04 | 0.772 | 31.97 | 8.13 | 35.94 | 6.79 | 32.91 | 10.90 | 0.505 | n/a | n/a | n/a |

**(Continued S5): Postural kinematics**

Kinematic abduction/ adduction joint angles recorded when standing square and in the stacked position (mean and inter-subject standard deviations) for the breed and each slope group. Normality p-values show if parameters are normally distributed (p>0.05). The correlation coefficient (r) and p-values show the correlation between the parameter and slope of the back (significant correlation highlighted (p<0.05)). When Kruskal-Wallis (KW) p-values were significant, Dunn posthoc values were calculated between the groups: levelled (Le), intermediate (In) and sloped (Sl). Values are rounded to two decimal places. The anatomical markers used to calculate each joint angle in the Sagittal plane are shown in brackets.

| **Abduction (positive) and adduction (negative) angle** | **Whole group** | | **Normality p-value** | **Correlation with slope** | | **Levelled** | | **Intermediate** | | **Sloped** | | **KW**  **p-value** | **Dunn posthoc** | | |
| --- | --- | --- | --- | --- | --- | --- | --- | --- | --- | --- | --- | --- | --- | --- | --- |
|  | **Mean** | **SD** |  | **r** | **p-value** | **Mean** | **SD** | **Mean** | **SD** | **Mean** | **SD** |  | **Le-In** | **Le-Sl** | **In-Sl** |
| **Standing square** | | | | | | | | | | | | | | | |
| Left Hip (IW-GT-LSF) | 25.35 | 8.10 | 0.668 | -0.02 | 0.899 | 24.64 | 6.63 | 26.19 | 7.22 | 25.27 | 8.67 | 0.729 | n/a | n/a | n/a |
| Right Hip (IW-GT-LSF) | 27.49 | 6.43 | 0.935 | 0.02 | 0.896 | 25.08 | 4.23 | 30.12 | 5.01 | 26.46 | 7.48 | 0.025 | 0.037 | 1.000 | 0.088 |
| Left Stifle (GT-LSF-LHK) | -13.27 | 8.10 | 0.449 | -0.36 | 0.004 | -10.04 | 5.20 | -10.74 | 4.98 | -16.52 | 9.23 | 0.046 | 1.000 | 0.065 | 0.195 |
| Right Stifle (GT-LSF-LHK) | -10.28 | 6.85 | 0.758 | -0.11 | 0.415 | -9.36 | 5.85 | -11.88 | 6.50 | -10.12 | 6.88 | 0.688 | n/a | n/a | n/a |
| Left Hock (LSF-LHK-MT) | 26.76 | 9.41 | 0.534 | 0.24 | 0.067 | 23.60 | 6.91 | 26.40 | 5.30 | 29.04 | 11.99 | 0.239 | n/a | n/a | n/a |
| Right Hock (LSF-LHK-MT) | 26.49 | 8.27 | 0.261 | 0.19 | 0.152 | 24.34 | 6.99 | 26.62 | 8.81 | 28.27 | 7.63 | 0.414 | n/a | n/a | n/a |
| **Stacked position** | | | | | | | | | | | | | | | |
| Left Hip (IW-GT-LSF) | 25.11 | 5.07 | 0.313 | -0.28 | 0.033 | 26.66 | 3.92 | 26.57 | 5.26 | 23.24 | 5.38 | 0.161 | n/a | n/a | n/a |
| Right Hip (IW-GT-LSF) | 26.30 | 7.05 | 0.105 | 0.16 | 0.225 | 23.43 | 5.22 | 28.58 | 7.35 | 27.21 | 7.84 | 0.091 | n/a | n/a | n/a |
| Left Stifle (GT-LSF-LHK) | -13.75 | 8.61 | 0.340 | -0.28 | 0.028 | -8.86 | 9.54 | -14.68 | 6.52 | -16.61 | 7.79 | 0.064 | n/a | n/a | n/a |
| Right Stifle (GT-LSF-LHK) | -10.46 | 6.20 | 0.180 | 0.09 | 0.503 | -11.57 | 6.06 | -10.35 | 7.51 | -9.63 | 5.38 | 0.627 | n/a | n/a | n/a |
| Left Hock (LSF-LHK-MT) | 25.62 | 10.15 | 0.862 | 0.20 | 0.119 | 21.43 | 11.88 | 25.82 | 7.49 | 28.57 | 10.10 | 0.187 | n/a | n/a | n/a |
| Right Hock (LSF-LHK-MT) | 29.12 | 9.24 | 0.592 | -0.02 | 0.874 | 29.63 | 10.94 | 27.96 | 9.58 | 29.65 | 7.63 | 0.999 | n/a | n/a | n/a |

**(Continued S5): Postural kinematics**

Limb postural parameters recorded from kinematic data when standing square (mean and inter-subject standard deviations) for the breed and each slope group. Normality p-values show if parameters are normally distributed (p>0.05). The correlation coefficient (r) and p-values show the correlation between the parameter and slope of the back (significant correlation highlighted (p<0.05)). When Kruskal-Wallis (KW) p-values were significant, Dunn posthoc values were calculated between the groups: levelled (Le), intermediate (In) and sloped (Sl). Values are rounded to two decimal places. (FR: Fore-right limb, FL: Fore-left limb, HR: Hind-right limb, HL: Hind-left limb).

| **Postural parameter** | **Whole group** | | **Normality p-value** | **Correlation with slope** | | **Levelled** | | **Intermediate** | | **Slope** | | **KW**  **p-value** | **Dunn posthoc** | | |
| --- | --- | --- | --- | --- | --- | --- | --- | --- | --- | --- | --- | --- | --- | --- | --- |
|  | **Mean** | **SD** |  | **r** | **p-value** | **Mean** | **SD** | **Mean** | **SD** | **Mean** | **SD** |  | **Le-In** | **Le-Sl** | **In-Sl** |
| **Limb position distances (% wither's height)** | | | | | | | | | | | | | | | |
| FR-HR | 92.58 | 9.26 | 0.624 | 0.17 | 0.192 | 90.97 | 10.69 | 93.15 | 7.54 | 93.56 | 9.77 | 0.560 | n/a | n/a | n/a |
| FL-HL | 97.02 | 8.18 | 0.508 | 0.07 | 0.617 | 97.32 | 6.71 | 95.74 | 6.32 | 98.06 | 10.79 | 0.636 | n/a | n/a | n/a |
| FR-FL | 36.56 | 4.61 | 0.853 | -0.58 | <0.001 | 40.17 | 3.79 | 35.59 | 3.80 | 34.03 | 4.09 | <0.001 | 0.003 | <0.001 | 1.000 |
| HR-HL | 40.91 | 5.69 | 0.722 | -0.32 | 0.012 | 41.75 | 5.11 | 41.95 | 5.81 | 39.15 | 5.90 | 0.363 | n/a | n/a | n/a |
| **Protraction (negative)/ retraction (positive) angle while standing** (°) | | | | | | | | | | | | | | | |
| FL | 7.72 | 5.05 | 0.580 | -0.09 | 0.470 | 7.98 | 6.03 | 8.27 | 3.79 | 6.86 | 5.49 | 0.461 | n/a | n/a | n/a |
| FR | 8.67 | 4.00 | 0.440 | -0.17 | 0.190 | 8.10 | 4.83 | 10.86 | 3.03 | 7.16 | 3.17 | 0.018 | 0.112 | 1.000 | 0.020 |
| HL | 8.55 | 4.25 | 0.420 | 0.56 | <0.001 | 6.10 | 2.19 | 8.43 | 3.13 | 10.71 | 5.26 | 0.004 | 0.126 | 0.003 | 0.727 |
| HR | 8.30 | 5.07 | 0.049 | 0.51 | <0.001 | 4.68 | 2.96 | 9.36 | 3.94 | 10.45 | 6.12 | 0.001 | 0.002 | 0.002 | 1.000 |

**(Continued S5): Postural kinematics**

| **Postural parameter** | **Whole group** | | **Normality p-value** | **Correlation with slope** | | **Levelled** | | **Intermediate** | | **Slope** | | **KW**  **p-value** | **Dunn posthoc** | | |
| --- | --- | --- | --- | --- | --- | --- | --- | --- | --- | --- | --- | --- | --- | --- | --- |
|  | **Mean** | **SD** |  | **r** | **p-value** | **Mean** | **SD** | **Mean** | **SD** | **Mean** | **SD** |  | **Le-In** | **Le-Sl** | **In-Sl** |
| **Segment lengths (% wither's height)** | | | | | | | | | | | | | | | |
| Withers-T8 | 19.43 | 4.49 | 0.185 | 0.18 | 0.166 | 17.93 | 5.48 | 20.12 | 3.99 | 20.15 | 3.79 | 0.401 | n/a | n/a | n/a |
| T8-L1 | 22.71 | 3.91 | 0.658 | 0.09 | 0.483 | 22.84 | 2.96 | 21.75 | 4.74 | 23.38 | 3.86 | 0.527 | n/a | n/a | n/a |
| L1-L5 | 19.55 | 2.71 | 0.338 | 0.05 | 0.701 | 20.14 | 2.71 | 18.61 | 2.66 | 19.87 | 2.65 | 0.187 | n/a | n/a | n/a |
| L5-Sacrum | 27.59 | 4.52 | 0.037 | -0.33 | 0.011 | 29.41 | 4.19 | 26.62 | 5.35 | 26.86 | 3.70 | 0.093 | n/a | n/a | n/a |
| Withers-Sacrum | 88.50 | 7.15 | 0.658 | -0.13 | 0.310 | 90.48 | 7.64 | 85.97 | 7.71 | 88.98 | 5.84 | 0.176 | n/a | n/a | n/a |
| Left Scapula | 25.95 | 3.87 | 0.083 | -0.02 | 0.908 | 25.76 | 4.20 | 26.94 | 3.81 | 24.59 | 3.46 | 0.379 | n/a | n/a | n/a |
| Right Scapula | 24.45 | 4.10 | 0.475 | 0.39 | 0.002 | 22.84 | 4.28 | 23.34 | 3.71 | 27.00 | 3.08 | 0.004 | 1.000 | 0.003 | 0.075 |
| Left Humerus | 27.90 | 3.96 | 0.569 | -0.43 | 0.0001 | 30.37 | 3.10 | 26.73 | 4.49 | 26.36 | 3.04 | 0.004 | 0.021 | 0.009 | 1.000 |
| Right Humerus | 27.50 | 3.14 | 0.266 | -0.09 | 0.506 | 27.44 | 3.08 | 28.49 | 3.93 | 26.68 | 2.23 | 0.161 | n/a | n/a | n/a |
| Left Radius | 34.71 | 2.49 | 0.313 | 0.17 | 0.197 | 34.43 | 1.66 | 34.65 | 3.42 | 35.00 | 2.18 | 0.667 | n/a | n/a | n/a |
| Right Radius | 35.28 | 2.08 | 0.575 | -0.12 | 0.373 | 35.88 | 1.78 | 34.90 | 2.64 | 35.10 | 1.65 | 0.290 | n/a | n/a | n/a |
| Left Metacarpals | 11.67 | 1.86 | 0.167 | -0.26 | 0.048 | 11.92 | 1.82 | 11.78 | 1.97 | 11.34 | 1.85 | 0.622 | n/a | n/a | n/a |
| Right Metacarpals | 11.27 | 1.54 | 0.510 | -0.33 | 0.010 | 11.79 | 1.67 | 11.33 | 1.25 | 10.69 | 1.54 | 0.150 | n/a | n/a | n/a |
| Left Pelvis | 27.06 | 2.67 | 0.650 | -0.17 | 0.189 | 27.22 | 3.77 | 27.22 | 2.56 | 26.87 | 2.40 | 0.880 | n/a | n/a | n/a |
| Right Pelvis | 28.45 | 2.60 | 0.972 | -0.37 | 0.003 | 29.36 | 2.86 | 28.99 | 2.11 | 27.11 | 2.39 | 0.038 | 1.000 | 0.062 | 0.114 |
| Left Femur | 32.08 | 3.12 | 0.677 | -0.13 | 0.339 | 32.26 | 3.32 | 32.39 | 2.86 | 31.73 | 3.28 | 0.998 | n/a | n/a | n/a |
| Right Femur | 33.07 | 3.47 | 0.495 | -0.06 | 0.665 | 34.06 | 2.82 | 32.34 | 4.61 | 32.84 | 2.73 | 0.529 | n/a | n/a | n/a |
| Left Tibia | 31.71 | 2.91 | 0.088 | -0.19 | 0.155 | 33.05 | 2.92 | 31.13 | 2.76 | 31.11 | 2.78 | 0.059 | n/a | n/a | n/a |
| Right Tibia | 30.40 | 3.11 | 0.771 | -0.09 | 0.506 | 30.59 | 3.18 | 30.70 | 3.93 | 30.00 | 2.35 | 0.644 | n/a | n/a | n/a |
| Left Metatarsals | 19.34 | 1.82 | 0.786 | -0.29 | 0.023 | 20.41 | 1.74 | 18.70 | 1.49 | 19.05 | 1.85 | 0.030 | 0.031 | 0.160 | 1.000 |
| Right Metatarsals | 18.72 | 2.16 | 0.014 | -0.39 | 0.002 | 19.90 | 2.59 | 18.21 | 1.52 | 18.11 | 1.88 | 0.053 | n/a | n/a | n/a |

**(Continued S5): Postural kinematics**

| **Postural parameter** | **Whole group** | | **Normality p-value** | **Correlation with slope** | | **Levelled** | | **Intermediate** | | **Slope** | | **KW**  **p-value** | **Dunn posthoc** | | |
| --- | --- | --- | --- | --- | --- | --- | --- | --- | --- | --- | --- | --- | --- | --- | --- |
|  | **Mean** | **SD** |  | **r** | **p-value** | **Mean** | **SD** | **Mean** | **SD** | **Mean** | **SD** |  | **Le-In** | **Le-Sl** | **In-Sl** |
| **Segment angle relative to anterior/ posterior horizontal (inclination of the segments) (°)** | | | | | | | | | | | | | | | |
| Left Scapula | 71.99 | 9.52 | 0.110 | 0.19 | 0.156 | 69.67 | 9.13 | 72.13 | 9.75 | 74.75 | 9.82 | 0.374 | n/a | n/a | n/a |
| Right Scapula | 73.09 | 9.30 | 0.153 | 0.06 | 0.628 | 73.31 | 8.90 | 67.80 | 10.08 | 77.38 | 7.02 | 0.060 | n/a | n/a | n/a |
| Left Humerus | 47.74 | 8.23 | 0.661 | -0.13 | 0.307 | 50.15 | 8.73 | 46.23 | 6.12 | 46.59 | 9.29 | 0.323 | n/a | n/a | n/a |
| Right Humerus | 47.18 | 7.09 | 0.073 | 0.03 | 0.835 | 48.63 | 7.57 | 44.51 | 6.32 | 48.23 | 7.02 | 0.166 | n/a | n/a | n/a |
| Left Radius | 84.98 | 2.92 | 0.025 | -0.02 | 0.873 | 85.83 | 2.10 | 84.45 | 3.64 | 84.74 | 2.78 | 0.431 | n/a | n/a | n/a |
| Right Radius | 84.48 | 3.09 | 0.224 | 0.13 | 0.321 | 84.74 | 2.91 | 83.41 | 2.42 | 85.23 | 3.64 | 0.061 | n/a | n/a | n/a |
| Left Metacarpals | 56.31 | 8.01 | 0.534 | -0.41 | 0.001 | 59.70 | 7.06 | 57.47 | 8.02 | 52.17 | 7.32 | 0.016 | 0.972 | 0.014 | 0.191 |
| Right Metacarpals | 60.74 | 9.02 | 0.056 | -0.17 | 0.195 | 62.52 | 7.95 | 60.02 | 9.18 | 59.72 | 9.99 | 0.676 | n/a | n/a | n/a |
| Left Pelvis | 39.19 | 6.97 | 0.934 | -0.06 | 0.637 | 43.80 | 5.38 | 36.49 | 7.77 | 39.40 | 6.02 | 0.091 | n/a | n/a | n/a |
| Right Pelvis | 36.23 | 7.28 | 0.726 | 0.10 | 0.449 | 35.43 | 9.25 | 35.24 | 4.97 | 37.98 | 7.24 | 0.501 | n/a | n/a | n/a |
| Left Femur | 82.61 | 4.21 | 0.005 | 0.34 | 0.009 | 79.68 | 3.62 | 83.82 | 4.05 | 83.72 | 3.85 | 0.001 | 0.004 | 0.004 | 1.000 |
| Right Femur | 81.56 | 4.10 | 0.515 | 0.18 | 0.164 | 80.68 | 4.64 | 81.63 | 3.91 | 82.24 | 3.89 | 0.484 | n/a | n/a | n/a |
| Left Tibia | 51.06 | 6.65 | 0.552 | -0.49 | <0.001 | 54.41 | 5.63 | 51.39 | 4.06 | 47.89 | 8.05 | 0.026 | 0.544 | 0.021 | 0.501 |
| Right Tibia | 52.48 | 8.17 | 0.701 | -0.47 | <0.001 | 56.21 | 7.16 | 53.74 | 5.22 | 48.12 | 9.17 | 0.008 | 1.000 | 0.008 | 0.145 |
| Left Metatarsals | 71.34 | 7.56 | 0.265 | 0.06 | 0.661 | 70.23 | 7.37 | 72.96 | 7.03 | 70.75 | 8.30 | 0.631 | n/a | n/a | n/a |
| Right Metatarsals | 70.76 | 7.48 | 0.168 | 0.11 | 0.398 | 68.99 | 9.29 | 72.90 | 5.22 | 70.29 | 7.33 | 0.291 | n/a | n/a | n/a |

**(Continued S5): Postural kinematics**

Kinematic limb postural parameters recorded in the stacked position (mean and inter-subject standard deviations) for the breed and each slope group. Normality p-values show if parameters are normally distributed (p>0.05). The correlation coefficient (r) and p-values show the correlation between the parameter and slope of the back (significant correlation highlighted (p<0.05)). When Kruskal-Wallis (KW) p-values were significant, Dunn posthoc values were calculated between the groups: levelled (Le), intermediate (In) and sloped (Sl). (FR: Fore-right limb, FL: Fore-left limb, HR: Hind-right limb, HL: Hind-left limb).

| **Postural parameter** | **Whole group** | | **Normality p-value** | **Correlation with slope** | | **Levelled** | | **Intermediate** | | **Slope** | | **KW**  **p-value** | **Dunn posthoc** | | |
| --- | --- | --- | --- | --- | --- | --- | --- | --- | --- | --- | --- | --- | --- | --- | --- |
|  | **Mean** | **SD** |  | **r** | **p-value** | **Mean** | **SD** | **Mean** | **SD** | **Mean** | **SD** |  | **Le-In** | **Le-Sl** | **In-Sl** |
| **Limb position distances (% wither's height)** | | | | | | | | | | | | | | | |
| FR-HR | 83.19 | 8.78 | 0.082 | -0.20 | 0.112 | 83.82 | 10.77 | 85.72 | 8.35 | 79.95 | 6.42 | 0.162 | n/a | n/a | n/a |
| FL-HL | 116.83 | 8.40 | 0.230 | -0.20 | 0.131 | 119.22 | 9.65 | 116.25 | 7.43 | 115.30 | 8.03 | 0.480 | n/a | n/a | n/a |
| FR-FL | 36.20 | 4.41 | 0.849 | -0.60 | <0.001 | 39.56 | 3.26 | 35.79 | 3.60 | 33.81 | 4.35 | 0.001 | 0.040 | 0.001 | 0.691 |
| HR-HL | 51.94 | 6.94 | 0.215 | -0.06 | 0.622 | 51.14 | 5.45 | 53.05 | 7.76 | 51.53 | 7.46 | 0.815 | n/a | n/a | n/a |
| **Protraction (negative)/ retraction (positive) angle** | | | | | | | | | | | | | | | |
| FL | 4.61 | 4.99 | 0.150 | 0.13 | 0.340 | 3.72 | 6.22 | 5.03 | 5.36 | 5.13 | 2.59 | 0.817 | n/a | n/a | n/a |
| FR | 6.83 | 3.49 | 0.150 | 0.06 | 0.630 | 6.33 | 3.34 | 6.23 | 3.21 | 7.69 | 3.85 | 0.545 | n/a | n/a | n/a |
| HL | 22.81 | 4.36 | 0.540 | 0.25 | 0.049 | 21.39 | 3.36 | 23.24 | 3.68 | 23.55 | 5.28 | 0.242 | n/a | n/a | n/a |
| HR | -4.04 | 5.71 | 0.250 | 0.16 | 0.23 | -4.28 | 7.25 | -4.19 | 4.57 | -3.69 | 5.53 | 0.470 | n/a | n/a | n/a |

**(Continued S5): Postural kinematics**

| **Postural parameter** | **Whole group** | | **Normality p-value** | **Correlation with slope** | | **Levelled** | | **Intermediate** | | **Slope** | | **KW**  **p-value** | **Dunn posthoc** | | |
| --- | --- | --- | --- | --- | --- | --- | --- | --- | --- | --- | --- | --- | --- | --- | --- |
|  | **Mean** | **SD** |  | **r** | **p-value** | **Mean** | **SD** | **Mean** | **SD** | **Mean** | **SD** |  | **Le-In** | **Le-Sl** | **In-Sl** |
| **Segment lengths (% wither's height)** | | | | | | | | | | | | | | | |
| Withers-T8 | 18.56 | 4.17 | 0.506 | 0.19 | 0.143 | 17.71 | 4.66 | 18.69 | 4.23 | 19.31 | 3.63 | 0.924 | n/a | n/a | n/a |
| T8-L1 | 23.08 | 4.30 | 0.446 | 0.02 | 0.893 | 23.16 | 3.67 | 22.86 | 5.20 | 23.20 | 4.15 | 0.489 | n/a | n/a | n/a |
| L1-L5 | 19.65 | 2.73 | 0.295 | 0.07 | 0.618 | 20.13 | 2.88 | 18.87 | 2.72 | 19.95 | 2.61 | 0.120 | n/a | n/a | n/a |
| L5-Sacrum | 27.36 | 4.45 | 0.049 | -0.34 | 0.009 | 29.33 | 4.48 | 26.06 | 4.75 | 26.82 | 3.68 | 0.026 | 0.102 | 0.032 | 1.000 |
| Withers-Sacrum | 87.61 | 7.47 | 0.742 | -0.13 | 0.339 | 89.91 | 9.06 | 84.87 | 7.67 | 88.08 | 4.16 | <0.001 | 0.154 | <0.001 | 0.139 |
| Left Scapula | 26.45 | 4.65 | 0.230 | -0.18 | 0.160 | 26.21 | 5.13 | 28.95 | 3.91 | 23.76 | 3.37 | 0.254 | n/a | n/a | n/a |
| Right Scapula | 24.31 | 3.43 | 0.194 | 0.58 | <0.001 | 21.21 | 0.97 | 24.73 | 2.19 | 26.35 | 3.55 | 0.290 | n/a | n/a | n/a |
| Left Humerus | 27.55 | 3.30 | 0.350 | -0.09 | 0.492 | 28.66 | 3.06 | 26.99 | 3.72 | 27.26 | 3.06 | 0.750 | n/a | n/a | n/a |
| Right Humerus | 27.19 | 2.77 | 0.290 | -0.10 | 0.438 | 27.81 | 3.03 | 27.51 | 2.84 | 26.30 | 2.38 | 0.536 | n/a | n/a | n/a |
| Left Radius | 34.69 | 2.39 | 0.913 | 0.13 | 0.325 | 34.51 | 1.99 | 34.53 | 3.22 | 34.99 | 1.89 | 0.608 | n/a | n/a | n/a |
| Right Radius | 35.24 | 2.09 | 0.859 | -0.03 | 0.843 | 35.34 | 1.61 | 35.20 | 2.93 | 35.19 | 1.52 | 0.839 | n/a | n/a | n/a |
| Left Metacarpals | 11.82 | 1.67 | 0.304 | -0.34 | 0.008 | 12.23 | 1.18 | 11.96 | 1.79 | 11.39 | 1.83 | 0.343 | n/a | n/a | n/a |
| Right Metacarpals | 11.16 | 1.53 | 0.333 | -0.34 | 0.008 | 11.79 | 1.74 | 11.21 | 1.22 | 10.60 | 1.50 | 0.566 | n/a | n/a | n/a |
| Left Pelvis | 26.77 | 2.75 | 0.674 | -0.21 | 0.107 | 27.62 | 2.68 | 26.26 | 3.06 | 26.58 | 2.62 | 0.003 | 0.114 | 0.002 | 0.587 |
| Right Pelvis | 28.09 | 2.59 | 0.333 | -0.25 | 0.053 | 28.27 | 2.88 | 28.89 | 2.38 | 27.21 | 2.44 | 0.020 | 0.312 | 0.016 | 0.685 |
| Left Femur | 31.25 | 3.26 | 0.814 | -0.09 | 0.517 | 31.69 | 3.65 | 30.92 | 2.68 | 31.10 | 3.37 | 0.854 | n/a | n/a | n/a |
| Right Femur | 33.70 | 3.20 | 0.760 | -0.16 | 0.222 | 34.55 | 2.25 | 33.64 | 4.72 | 33.00 | 2.13 | 0.263 | n/a | n/a | n/a |
| Left Tibia | 33.88 | 3.13 | 0.066 | -0.23 | 0.071 | 34.40 | 3.29 | 34.35 | 3.37 | 33.09 | 2.77 | 0.417 | n/a | n/a | n/a |
| Right Tibia | 29.69 | 2.77 | 0.574 | -0.20 | 0.133 | 30.51 | 2.83 | 29.20 | 3.49 | 29.38 | 1.90 | 0.566 | n/a | n/a | n/a |
| Left Metatarsals | 19.85 | 1.93 | 0.354 | -0.38 | 0.003 | 21.20 | 1.42 | 19.28 | 1.77 | 19.32 | 1.96 | 0.005 | 0.012 | 0.012 | 1.000 |
| Right Metatarsals | 18.62 | 2.22 | 0.092 | -0.30 | 0.022 | 19.36 | 2.56 | 18.40 | 1.88 | 18.23 | 2.19 | 0.357 | n/a | n/a | n/a |

**(Continued S5): Postural kinematics**

| **Postural parameter** | **Whole group** | | **Normality p-value** | **Correlation with slope** | | **Levelled** | | **Intermediate** | | **Slope** | | **KW**  **p-value** | **Dunn posthoc** | | |
| --- | --- | --- | --- | --- | --- | --- | --- | --- | --- | --- | --- | --- | --- | --- | --- |
|  | **Mean** | **SD** |  | **r** | **p-value** | **Mean** | **SD** | **Mean** | **SD** | **Mean** | **SD** |  | **Le-In** | **Le-Sl** | **In-Sl** |
| **Segment angle relative to horizontal (°)** | | | | | | | | | | | | | | | |
| Left Scapula | 72.11 | 9.85 | 0.419 | 0.24 | 0.062 | 72.00 | 9.77 | 67.30 | 9.94 | 78.66 | 6.15 | 0.018 | 0.693 | 0.282 | 0.013 |
| Right Scapula | 70.46 | 10.07 | 0.470 | 0.17 | 0.182 | 69.12 | 10.21 | 64.46 | 9.29 | 74.64 | 8.99 | 0.068 | n/a | n/a | n/a |
| Left Humerus | 50.08 | 8.63 | 0.082 | -0.15 | 0.247 | 53.08 | 8.75 | 48.63 | 7.47 | 49.23 | 9.68 | 0.512 | n/a | n/a | n/a |
| Right Humerus | 51.43 | 6.32 | 0.258 | -0.13 | 0.320 | 52.74 | 6.34 | 50.60 | 4.70 | 50.99 | 7.73 | 0.312 | n/a | n/a | n/a |
| Left Radius | 84.95 | 2.47 | 0.428 | 0.24 | 0.062 | 84.46 | 2.67 | 84.46 | 2.44 | 85.84 | 2.20 | 0.151 | n/a | n/a | n/a |
| Right Radius | 85.49 | 2.33 | 0.001 | 0.12 | 0.372 | 85.54 | 1.88 | 85.00 | 2.73 | 85.96 | 2.31 | 0.440 | n/a | n/a | n/a |
| Left Metacarpals | 53.44 | 9.26 | 0.745 | -0.20 | 0.125 | 54.77 | 8.83 | 55.20 | 10.07 | 50.69 | 8.70 | 0.382 | n/a | n/a | n/a |
| Right Metacarpals | 58.10 | 8.99 | 0.939 | 0.00 | 0.989 | 60.18 | 7.65 | 53.59 | 8.74 | 60.53 | 9.15 | 0.061 | n/a | n/a | n/a |
| Left Pelvis | 38.37 | 7.48 | 0.207 | 0.15 | 0.253 | 38.01 | 8.59 | 36.53 | 10.06 | 39.78 | 4.48 | 0.723 | n/a | n/a | n/a |
| Right Pelvis | 37.51 | 6.33 | 0.338 | 0.22 | 0.092 | 36.74 | 7.10 | 35.82 | 6.22 | 39.67 | 5.56 | 0.235 | n/a | n/a | n/a |
| Left Femur | 83.41 | 3.39 | 0.048 | -0.18 | 0.173 | 84.13 | 2.75 | 83.17 | 2.95 | 83.02 | 4.06 | 0.682 | n/a | n/a | n/a |
| Right Femur | 75.81 | 6.43 | 0.304 | 0.04 | 0.771 | 76.30 | 7.35 | 75.53 | 6.58 | 75.64 | 5.83 | 0.917 | n/a | n/a | n/a |
| Left Tibia | 37.14 | 5.72 | 0.791 | -0.29 | 0.024 | 39.07 | 4.25 | 37.14 | 4.75 | 35.53 | 7.21 | 0.202 | n/a | n/a | n/a |
| Right Tibia | 62.14 | 7.94 | 0.385 | -0.29 | 0.025 | 64.34 | 7.90 | 62.33 | 5.99 | 60.03 | 9.16 | 0.273 | n/a | n/a | n/a |
| Left Metatarsals | 82.16 | 4.70 | 0.006 | 0.05 | 0.730 | 80.16 | 5.52 | 83.76 | 3.46 | 82.23 | 4.65 | 0.136 | n/a | n/a | n/a |
| Right Metatarsals | 55.62 | 8.95 | 0.134 | -0.08 | 0.548 | 56.42 | 8.11 | 56.22 | 9.07 | 54.32 | 9.80 | 0.518 | n/a | n/a | n/a |

**S6: Trotting Kinetics**

Kinetic parameters normalised to body weight, recorded during trot (mean and inter-subject standard deviations) for the breed and each slope group. Normality p-values show if parameters are normally distributed (p>0.05). The correlation coefficient (r) and p-values show the correlation between the parameter and slope of the back (significant correlation highlighted (p<0.05)). When Kruskal-Wallis (KW) p-values were significant, Dunn posthoc values were calculated between the groups: levelled (Le), intermediate (In) and sloped (Sl). (FL: forelimb, HL: hind limb, BW: body weight, met: metacarpal/ metatarsal pad, dig: digital pads).

| **Kinetic parameter** | **Whole group** | | **Normality p-value** | **Correlation with slope** | | **Levelled** | | | | **Intermediate** | | **Sloped** | | **KW p-value** | **Dunn posthoc** | | |
| --- | --- | --- | --- | --- | --- | --- | --- | --- | --- | --- | --- | --- | --- | --- | --- | --- | --- |
|  | **Mean** | **SD** |  | **r** | **p-value** | **Mean** | | **SD** | | **Mean** | **SD** | **Mean** | **SD** |  | **Le-In** | **Le-Sl** | **In-Sl** |
| Right FL % Weight bearing | 50.06 | 1.35 | 0.697 | 0.14 | 0.286 | 49.84 | | 1.45 | | 50.13 | 1.26 | 50.18 | 1.39 | 0.746 | n/a | n/a | n/a |
| Left FL % Weight bearing | 49.94 | 1.35 | 0.697 | -0.14 | 0.286 | 50.16 | | 1.45 | | 49.87 | 1.26 | 49.82 | 1.39 | 0.746 | n/a | n/a | n/a |
| Right HL % Weight bearing | 49.53 | 1.36 | 0.145 | 0.03 | 0.847 | 49.58 | | 0.92 | | 49.73 | 1.37 | 49.30 | 1.69 | 0.569 | n/a | n/a | n/a |
| Left HL % Weight bearing | 50.47 | 1.36 | 0.145 | -0.03 | 0.847 | 50.42 | | 0.92 | | 50.27 | 1.37 | 50.70 | 1.69 | 0.569 | n/a | n/a | n/a |
| FL % Weight bearing | 62.69 | 2.40 | 0.944 | 0.26 | 0.045 | 61.50 | | 2.30 | | 63.64 | 2.30 | 62.76 | 2.23 | 0.006 | 0.005 | 0.091 | 0.935 |
| HL % Weight bearing | 37.31 | 2.40 | 0.944 | -0.26 | 0.045 | 38.50 | | 2.30 | | 36.36 | 2.30 | 37.24 | 2.23 | 0.006 | 0.005 | 0.091 | 0.935 |
| **Right Forelimb** | | | | | | | | | | | | | | | | | |
| Vertical force (%BW) | 120.68 | 17.83 | 0.721 | 0.58 | <0.001 | 106.62 | | 13.15 | | 123.84 | 15.87 | 128.92 | 16.77 | <0.001 | 0.009 | <0.001 | 1.000 |
| Contact area (cm^2^/kg) | 0.81 | 0.11 | 0.143 | 0.52 | <0.001 | 0.75 | | 0.09 | | 0.79 | 0.09 | 0.87 | 0.12 | 0.004 | 0.559 | 0.003 | 0.127 |
| Vertical force_dig (%BW) | 57.39 | 10.19 | 0.090 | 0.50 | <0.001 | 50.33 | | 8.85 | | 58.83 | 9.25 | 61.87 | 9.35 | 0.003 | 0.012 | 0.001 | 1.000 |
| Vertical force_met (%BW) | 62.75 | 12.05 | 0.313 | 0.38 | 0.003 | 56.29 | | 10.82 | | 65.01 | 11.10 | 65.73 | 12.41 | 0.036 | 0.103 | 0.050 | 1.000 |
| Contact area_dig (cm^2^/kg) | 0.42 | 0.09 | 0.099 | 0.16 | 0.234 | 0.39 | | 0.11 | | 0.43 | 0.08 | 0.43 | 0.10 | 0.297 | n/a | n/a | n/a |
| Contact area_met (cm^2^/kg) | 0.37 | 0.07 | 0.001 | 0.40 | 0.001 | 0.34 | | 0.07 | | 0.37 | 0.06 | 0.40 | 0.07 | 0.029 | 0.644 | 0.024 | 0.422 |
| Peak vertical force_dig (%BW) | 11.35 | 2.12 | 0.419 | 0.32 | 0.011 | 10.63 | | 1.69 | | 11.38 | 1.86 | 11.92 | 2.57 | 0.136 | n/a | n/a | n/a |
| Peak vertical force_met (%BW) | 11.54 | 2.81 | 0.434 | 0.24 | 0.067 | 10.88 | | 2.75 | | 11.47 | 2.90 | 12.19 | 2.76 | 0.362 | n/a | n/a | n/a |
| Impulse (kg.s) | 6.72 | 1.06 | 0.751 | 0.30 | 0.022 | 6.47 | | 0.88 | | 6.66 | 1.26 | 7.00 | 0.96 | 0.206 | n/a | n/a | n/a |
| Max peak pressure (kPa) | 483.04 | 60.19 | 0.536 | -0.02 | 0.869 | 493.59 | | 44.69 | | 475.46 | 61.27 | 481.59 | 71.22 | 0.429 | n/a | n/a | n/a |
| **Left Forelimb** | | | | | | | | | | | | | | | | | |
| Vertical force (%BW) | 121.24 | 16.93 | 0.357 | 0.55 | <0.001 | 107.26 | | 10.11 | | 125.77 | 12.89 | 128.24 | 18.41 | <0.001 | 0.001 | <0.001 | 1.000 |
| Contact area (cm^2^/kg) | 0.82 | 0.12 | 0.035 | 0.49 | <0.001 | 0.75 | | 0.08 | | 0.82 | 0.13 | 0.88 | 0.11 | 0.002 | 0.206 | 0.001 | 0.187 |
| Vertical force_dig (%BW) | 57.19 | 10.61 | 0.633 | 0.59 | <0.001 | 49.18 | | 6.33 | | 57.20 | 8.22 | 63.68 | 11.33 | <0.001 | 0.022 | <0.001 | 0.274 |
| Vertical force_met (%BW) | 64.35 | 12.27 | 0.360 | 0.29 | 0.024 | 59.91 | | 12.15 | | 66.62 | 10.74 | 65.98 | 13.37 | 0.128 | n/a | n/a | n/a |
| Contact area_dig (cm^2^/kg) | 0.41 | 0.10 | 0.014 | 0.18 | 0.172 | 0.38 | | 0.10 | | 0.42 | 0.08 | 0.43 | 0.11 | 0.183 | n/a | n/a | n/a |
| Contact area_met (cm^2^/kg) | 0.37 | 0.06 | 0.012 | 0.46 | <0.001 | 0.33 | | 0.04 | | 0.37 | 0.06 | 0.39 | 0.07 | 0.010 | 0.216 | 0.007 | 0.546 |
| Peak vertical force_dig (%BW) | 11.21 | 1.57 | 0.116 | 0.29 | 0.025 | 10.66 | | 1.24 | | 11.23 | 1.59 | 11.67 | 1.73 | 0.025 | 0.501 | 0.020 | 0.464 |
| Peak vertical force_met (%BW) | 12.24 | 3.03 | 0.835 | 0.04 | 0.744 | 12.18 | | 2.27 | | 12.48 | 3.29 | 12.05 | 3.43 | 0.951 | n/a | n/a | n/a |
| Impulse (kg.s) | 6.83 | 1.15 | 0.763 | 0.26 | 0.046 | 6.63 | | 1.04 | | 6.73 | 1.30 | 7.12 | 1.07 | 0.298 | n/a | n/a | n/a |
| Max peak pressure (kPa) | 496.54 | 76.09 | 0.255 | -0.06 | 0.641 | 512.17 | | 65.49 | | 487.63 | 74.99 | 492.04 | 86.51 | 0.468 | n/a | n/a | n/a |
| **Right Hind limb** | | | | | | | | | | | | | | | | | |
| Vertical force (%BW) | 70.41 | 8.66 | 0.174 | 0.42 | 0.001 | 66.17 | | 7.49 | | 70.54 | 9.35 | 73.88 | 7.54 | 0.026 | 0.292 | 0.022 | 0.807 |
| Contact area (cm^2^/kg) | 0.59 | 0.08 | 0.135 | 0.33 | 0.010 | 0.57 | | 0.07 | | 0.58 | 0.09 | 0.62 | 0.08 | 0.107 | n/a | n/a | n/a |
| Vertical force_dig (%BW) | 51.27 | 8.41 | 0.100 | 0.62 | <0.001 | 44.05 | | 6.14 | | 52.61 | 6.93 | 56.01 | 7.58 | <0.001 | 0.009 | <0.001 | 0.476 |
| Vertical force_met (%BW) | 19.42 | 5.25 | 0.797 | -0.27 | 0.040 | 21.79 | | 3.75 | | 18.70 | 5.28 | 18.20 | 5.82 | 0.052 | n/a | n/a | n/a |
| Contact area_dig (cm^2^/kg) | 0.40 | 0.06 | 0.078 | 0.48 | <0.001 | 0.36 | | 0.05 | | 0.39 | 0.06 | 0.43 | 0.06 | 0.010 | 0.602 | 0.008 | 0.195 |
| Contact area_met (cm^2^/kg) | 0.19 | 0.04 | 0.498 | 0.04 | 0.784 | 0.19 | | 0.03 | | 0.18 | 0.04 | 0.19 | 0.05 | 0.762 | n/a | n/a | n/a |
| Peak vertical force_dig (%BW) | 11.11 | 2.55 | <0.001 | 0.31 | 0.014 | 10.61 | | 2.32 | | 10.86 | 2.43 | 11.67 | 2.82 | 0.503 | n/a | n/a | n/a |
| Peak vertical force_met (%BW) | 6.41 | 1.78 | 0.787 | -0.18 | 0.164 | 6.97 | | 1.55 | | 5.99 | 1.65 | 6.34 | 2.03 | 0.182 | n/a | n/a | n/a |
| Impulse (kg.s) | 3.87 | 0.61 | 0.464 | 0.09 | 0.491 | 3.96 | | 0.57 | | 3.67 | 0.47 | 3.98 | 0.73 | 0.255 | n/a | n/a | n/a |
| Max peak pressure (kPa) | 417.04 | 53.02 | 0.492 | 0.24 | 0.060 | 403.69 | | 41.82 | | 425.27 | 54.49 | 420.25 | 59.96 | 0.405 | n/a | n/a | n/a |
| **Left Hind limb** | | | | | | | | | | | | | | | | | |
| Vertical force (%BW) | 71.47 | 8.88 | 0.310 | 0.37 | 0.004 | 67.59 | 8.59 | | 70.72 | 9.20 | 75.56 | 7.32 | | 0.019 | 0.797 | 0.016 | 0.229 |
| Contact area (cm^2^/kg) | 0.61 | 0.09 | 0.081 | 0.28 | 0.030 | 0.59 | 0.09 | | 0.59 | 0.09 | 0.63 | 0.09 | | 0.107 | n/a | n/a | n/a |
| Vertical force_dig (%BW) | 53.05 | 8.59 | 0.284 | 0.55 | <0.001 | 46.60 | 6.81 | | 53.72 | 6.81 | 57.59 | 8.54 | | <0.001 | 0.017 | <0.001 | 0.718 |
| Vertical force_met (%BW) | 18.70 | 5.72 | 0.813 | -0.23 | 0.079 | 21.25 | 3.57 | | 17.00 | 6.09 | 18.20 | 6.31 | | 0.024 | 0.020 | 0.975 | 0.248 |
| Contact area_dig (cm^2^/kg) | 0.41 | 0.07 | 0.070 | 0.32 | 0.012 | 0.40 | 0.06 | | 0.40 | 0.06 | 0.43 | 0.07 | | 0.085 | n/a | n/a | n/a |
| Contact area_met (cm^2^/kg) | 0.19 | 0.04 | 0.786 | 0.02 | 0.870 | 0.19 | 0.04 | | 0.19 | 0.03 | 0.19 | 0.05 | | 0.924 | n/a | n/a | n/a |
| Peak vertical force_dig (%BW) | 10.87 | 1.94 | 0.113 | 0.24 | 0.064 | 10.26 | 1.81 | | 11.29 | 2.07 | 10.95 | 1.85 | | 0.297 | n/a | n/a | n/a |
| Peak vertical force_met (%BW) | 6.51 | 2.13 | 0.388 | -0.11 | 0.385 | 7.24 | 1.59 | | 5.44 | 1.45 | 6.87 | 2.68 | | 0.017 | 0.016 | 0.949 | 0.177 |
| Impulse (kg.s) | 3.95 | 0.61 | 0.626 | 0.09 | 0.485 | 4.06 | 0.55 | | 3.68 | 0.50 | 4.11 | 0.71 | | 0.074 | n/a | n/a | n/a |
| Max peak pressure (kPa) | 424.75 | 55.48 | 0.408 | 0.05 | 0.678 | 426.48 | 49.38 | | 428.13 | 61.75 | 419.89 | 56.16 | | 0.916 | n/a | n/a | n/a |

**(Continued S6): Trotting kinetics**

Stride parameters normalised to body weight, stride time and length of the back where necessary, recorded during trot (mean and inter-subject standard deviations) for the breed and each slope group. Normality p-values show if parameters are normally distributed (p>0.05). The correlation coefficient (r) and p-values show the correlation between the parameter and slope of the back (significant correlation highlighted (p<0.05)). When Kruskal-Wallis (KW) p-values were significant, Dunn posthoc values were calculated between the groups: levelled (Le), intermediate (In) and sloped (Sl).

| **Stride parameter** | **Whole group** | | **Normality p-value** | **Correlation with slope** | | **Levelled** | | **Intermediate** | | **Sloped** | | **KW**  **p-value** | **Dunn posthoc** | | |
| --- | --- | --- | --- | --- | --- | --- | --- | --- | --- | --- | --- | --- | --- | --- | --- |
|  | **Mean** | **SD** |  | **r** | **p-value** | **Mean** | **SD** | **Mean** | **SD** | **Mean** | **SD** |  | **Le-In** | **Le-Sl** | **In-Sl** |
| Stride time (sec) | 0.53 | 0.03 | 0.465 | 0.32 | 0.014 | 0.53 | 0.03 | 0.53 | 0.03 | 0.54 | 0.04 | 0.268 |  |  |  |
| Stride length (% wither’s height) | 204.94 | 17.02 | 0.001 | 0.16 | 0.231 | 197.73 | 10.18 | 208.85 | 24.18 | 207.19 | 10.65 | 0.048 | 0.237 | 0.049 | 1.000 |
| Stride speed (cm/sec) | 243.27 | 22.55 | 0.003 | 0.22 | 0.096 | 239.40 | 13.15 | 246.13 | 28.88 | 243.56 | 21.88 | 0.947 |  |  |  |
| **Right forelimb** | | | | | | | | | | | | | | | |
| Stance time (% stride time) | 45.64 | 2.60 | 0.903 | -0.16 | 0.235 | 46.45 | 2.50 | 45.26 | 2.64 | 45.32 | 2.62 | 0.233 | n/a | n/a | n/a |
| Swing time (% stride time) | 54.47 | 2.57 | 0.447 | 0.14 | 0.293 | 53.79 | 2.44 | 54.73 | 2.58 | 54.78 | 2.68 | 0.298 | n/a | n/a | n/a |
| **Right hind limb** | | | | | | | | | | | | | | | |
| Stance time (% stride time) | 42.96 | 3.03 | 0.917 | -0.31 | 0.017 | 44.70 | 3.32 | 42.05 | 2.75 | 42.38 | 2.49 | 0.018 | 0.046 | 0.035 | 1.000 |
| Swing time (% stride time) | 57.77 | 2.83 | 0.862 | 0.34 | 0.007 | 56.20 | 2.89 | 58.40 | 2.77 | 58.47 | 2.40 | 0.021 | 0.071 | 0.030 | 1.000 |
| **Left forelimb** | | | | | | | | | | | | | | | |
| Stance time (% stride time) | 46.07 | 2.55 | 0.717 | -0.22 | 0.090 | 47.47 | 2.45 | 45.00 | 2.53 | 45.94 | 2.18 | 0.005 | 0.003 | 0.140 | 0.546 |
| Swing time (% stride time) | 54.03 | 2.48 | 0.962 | 0.18 | 0.170 | 52.85 | 2.49 | 54.93 | 2.41 | 54.14 | 2.21 | 0.032 | 0.026 | 0.373 | 0.774 |
| **Left hind limb** | | | | | | | | | | | | | | | |
| Stance time (% stride time) | 42.98 | 3.11 | 0.884 | -0.25 | 0.057 | 44.53 | 2.48 | 41.76 | 2.85 | 42.86 | 3.39 | 0.012 | 0.010 | 0.123 | 1.000 |
| Swing time (% stride time) | 57.64 | 3.09 | 0.945 | 0.27 | 0.034 | 56.18 | 2.69 | 58.59 | 2.86 | 57.94 | 3.31 | 0.023 | 0.031 | 0.086 | 1.000 |

**S7: Trotting Kinematics**

Kinematic flexion/ extension joint and segment angles recorded during trot (mean and inter-subject standard deviations) of the minimum and maximum joint angles for the breed and each slope group. Normality p-values show if parameters are normally distributed (p>0.05). The correlation coefficient (r) and p-values show the correlation between the parameter and slope of the back (significant correlation highlighted (p<0.05)). When Kruskal-Wallis (Fr) p-values were significant, Dunn posthoc values were calculated between the groups: levelled (Le), intermediate (In) and sloped (Sl). Values are rounded to two decimal places. The anatomical markers used to calculate each joint angle in the Sagittal plane are shown in brackets. (FR: Fore-right limb, FL: Fore-left limb, HR: Hind-right limb, HL: Hind-left limb).

| **Minimum flexion (positive) angle or maximum extension (negative) angle** | **Whole group** | | **Normality p-value** | **Correlation with slope** | | **Levelled** | | **Intermediate** | | **Sloped** | | **KW**  **p-value** | **Dunn posthoc** | | |
| --- | --- | --- | --- | --- | --- | --- | --- | --- | --- | --- | --- | --- | --- | --- | --- |
|  | **Mean** | **SD** |  | **r** | **p-value** | **Mean** | **SD** | **Mean** | **SD** | **Mean** | **SD** |  | **Le-In** | **Le-Sl** | **In-Sl** |
| Neck (Head-with-T8) | -9.75 | 8.92 | 0.175 | -0.26 | 0.047 | -8.11 | 2.98 | -2.85 | 7.55 | -11.90 | 9.35 | 0.130 | n/a | n/a | n/a |
| Mid Thorax (With-T8-L1) | -5.51 | 5.22 | 0.572 | 0.26 | 0.046 | -8.92 | 3.25 | -5.47 | 5.57 | -4.34 | 4.84 | 0.123 | n/a | n/a | n/a |
| Thoraco-lumbar (T8-L1-L5) | -1.79 | 6.86 | 0.595 | 0.23 | 0.079 | -5.71 | 3.88 | -4.33 | 7.84 | -0.04 | 6.47 | 0.103 | n/a | n/a | n/a |
| Lumbo-sacral (L1-L5-Sacr) | 8.03 | 3.74 | 0.352 | -0.18 | 0.163 | 10.09 | 3.09 | 7.73 | 2.27 | 7.79 | 4.13 | 0.317 | n/a | n/a | n/a |
| Tail set (L5-Sacr-Co3) | 11.26 | 16.49 | <0.001 | -0.08 | 0.545 | 17.54 | 9.63 | 6.44 | 17.62 | 11.79 | 16.07 | 0.458 | n/a | n/a | n/a |
| Co3 (Sacr-Co3-Co8) | -2.72 | 14.84 | 0.017 | -0.16 | 0.234 | 9.96 | 7.70 | -8.56 | 18.40 | -2.81 | 11.03 | 0.043 | 0.063 | 0.073 | 1.000 |
| Co8 (Co3-Co8-Co14) | -16.60 | 21.65 | 0.392 | -0.22 | 0.095 | -7.07 | 12.93 | -21.39 | 22.42 | -18.16 | 20.65 | 0.303 | n/a | n/a | n/a |
| Left Hip (IW-GT-LSF) | 7.79 | 7.98 | 0.356 | -0.06 | 0.650 | 14.48 | 6.42 | 4.40 | 7.90 | 8.32 | 7.64 | 0.092 | n/a | n/a | n/a |
| Right Hip (IW-GT-LSF) | 11.81 | 13.07 | 0.219 | -0.05 | 0.683 | 12.02 | 14.06 | 16.14 | 16.05 | 9.74 | 9.10 | 0.284 | n/a | n/a | n/a |
| Left Stifle (GT-LSF-LHK) | 26.87 | 9.12 | 0.295 | 0.19 | 0.155 | 29.42 | 8.84 | 21.87 | 8.06 | 28.90 | 8.06 | 0.171 | n/a | n/a | n/a |
| Right Stifle (GT-LSF-LHK) | 26.00 | 8.28 | 0.591 | 0.08 | 0.538 | 24.71 | 9.46 | 22.41 | 7.87 | 28.13 | 6.85 | 0.340 | n/a | n/a | n/a |
| Left Hock (LSF-LHK-MT) | 37.23 | 6.26 | 0.473 | 0.31 | 0.017 | 33.68 | 3.80 | 36.21 | 3.85 | 38.60 | 6.99 | 0.274 | n/a | n/a | n/a |
| Right Hock (LSF-LHK-MT) | 34.57 | 8.35 | 0.008 | 0.13 | 0.304 | 33.36 | 10.25 | 33.78 | 4.27 | 35.39 | 8.71 | 0.501 | n/a | n/a | n/a |
| Left Shoulder (TS-AA-LEB) | 39.93 | 14.99 | 0.553 | 0.01 | 0.935 | 39.55 | 7.75 | 41.94 | 12.78 | 40.66 | 18.43 | 0.888 | n/a | n/a | n/a |
| Right Shoulder (TS-AA-LEB) | 38.90 | 5.72 | 0.090 | -0.13 | 0.309 | 36.30 | 7.34 | 41.99 | 4.07 | 36.46 | 6.45 | 0.366 | n/a | n/a | n/a |
| Left Elbow (HH-LEB-LCP) | 40.75 | 9.03 | 0.468 | 0.03 | 0.816 | 41.46 | 5.64 | 41.24 | 6.16 | 40.53 | 10.91 | 0.998 | n/a | n/a | n/a |
| Right Elbow (HH-LEB-LCP) | 34.99 | 8.59 | 0.529 | -0.05 | 0.691 | 35.75 | 5.16 | 32.77 | 5.28 | 36.23 | 10.75 | 0.507 | n/a | n/a | n/a |
| Left Carpal (LEB-LCP-MC) | -51.27 | 10.15 | 0.603 | -0.31 | 0.015 | -45.47 | 10.88 | -53.66 | 9.26 | -52.92 | 9.90 | 0.309 | n/a | n/a | n/a |
| Right Carpal (LEB-LCP-MC) | -50.79 | 12.33 | 0.037 | 0.00 | 0.984 | -43.93 | 3.99 | -57.32 | 11.34 | -50.01 | 13.39 | 0.092 | n/a | n/a | n/a |

**(Continued S7): Trotting kinematics**

| **Maximum flexion (positive) angle or minimum extension (negative) angle** | **Whole group** | | **Normality p-value** | **Correlation with slope** | | **Levelled** | | **Intermediate** | | **Sloped** | | **KW**  **p-value** | **Dunn posthoc** | | |
| --- | --- | --- | --- | --- | --- | --- | --- | --- | --- | --- | --- | --- | --- | --- | --- |
|  | **Mean** | **SD** |  | **r** | **p-value** | **Mean** | **SD** | **Mean** | **SD** | **Mean** | **SD** |  | **Le-In** | **Le-Sl** | **In-Sl** |
| Neck (Head-with-T8) | 4.14 | 7.97 | 0.701 | -0.11 | 0.417 | 3.59 | 3.99 | 8.40 | 5.24 | 3.32 | 9.15 | 0.354 | n/a | n/a | n/a |
| Mid Thorax (With-T8-L1) | 7.06 | 3.98 | 0.979 | 0.38 | 0.003 | 3.63 | 2.41 | 7.68 | 5.03 | 7.94 | 3.44 | 0.057 | n/a | n/a | n/a |
| Thoraco-lumbar (T8-L1-L5) | 8.72 | 5.76 | 0.171 | 0.25 | 0.051 | 5.62 | 4.30 | 7.39 | 6.42 | 9.91 | 5.48 | 0.132 | n/a | n/a | n/a |
| Lumbo-sacral (L1-L5-Sacr) | 14.68 | 3.45 | 0.989 | -0.21 | 0.116 | 16.51 | 3.32 | 14.67 | 1.92 | 14.37 | 3.70 | 0.454 | n/a | n/a | n/a |
| Tail set (L5-Sacr-Co3) | 18.37 | 16.83 | <0.001 | -0.06 | 0.646 | 23.97 | 8.97 | 13.22 | 18.38 | 19.07 | 16.37 | 0.757 | n/a | n/a | n/a |
| Co3 (Sacr-Co3-Co8) | 10.68 | 14.28 | 0.012 | -0.34 | 0.008 | 24.73 | 4.47 | 6.25 | 15.04 | 8.66 | 12.96 | 0.006 | 0.020 | 0.008 | 1.000 |
| Co8 (Co3-Co8-Co14) | -1.52 | 21.80 | 0.652 | -0.16 | 0.228 | 9.10 | 14.93 | -6.72 | 20.97 | -2.50 | 21.73 | 0.253 | n/a | n/a | n/a |
| Left Hip (IW-GT-LSF) | 54.11 | 8.50 | 0.752 | -0.09 | 0.484 | 60.23 | 11.01 | 50.79 | 8.96 | 54.06 | 5.52 | 0.290 | n/a | n/a | n/a |
| Right Hip (IW-GT-LSF) | 58.58 | 10.75 | 0.467 | 0.01 | 0.919 | 56.21 | 14.29 | 65.48 | 7.38 | 55.97 | 8.84 | 0.070 | n/a | n/a | n/a |
| Left Stifle (GT-LSF-LHK) | 88.64 | 9.24 | 0.966 | 0.18 | 0.180 | 89.40 | 9.20 | 87.40 | 9.44 | 89.41 | 8.59 | 0.961 | n/a | n/a | n/a |
| Right Stifle (GT-LSF-LHK) | 90.53 | 8.50 | 0.370 | 0.36 | 0.005 | 85.01 | 5.56 | 87.27 | 7.04 | 94.05 | 7.91 | 0.034 | 1.000 | 0.061 | 0.171 |
| Left Hock (LSF-LHK-MT) | 99.79 | 9.02 | 0.351 | 0.25 | 0.054 | 96.08 | 10.84 | 99.85 | 8.26 | 100.76 | 8.11 | 0.610 | n/a | n/a | n/a |
| Right Hock (LSF-LHK-MT) | 99.87 | 12.89 | 0.431 | 0.36 | 0.005 | 93.24 | 12.91 | 95.83 | 13.25 | 104.00 | 10.66 | 0.177 | n/a | n/a | n/a |
| Left Shoulder (TS-AA-LEB) | 70.00 | 13.88 | 0.390 | 0.15 | 0.257 | 68.07 | 8.67 | 70.39 | 11.31 | 72.26 | 16.78 | 0.828 | n/a | n/a | n/a |
| Right Shoulder (TS-AA-LEB) | 73.57 | 8.73 | 0.065 | 0.23 | 0.076 | 68.21 | 8.58 | 79.73 | 4.41 | 72.42 | 7.51 | 0.027 | 0.025 | 1.000 | 0.191 |
| Left Elbow (HH-LEB-LCP) | 108.03 | 7.75 | 0.679 | 0.27 | 0.035 | 105.19 | 5.66 | 108.30 | 5.82 | 109.29 | 8.46 | 0.405 | n/a | n/a | n/a |
| Right Elbow (HH-LEB-LCP) | 103.25 | 10.05 | 0.161 | 0.34 | 0.008 | 100.38 | 5.01 | 100.68 | 6.63 | 106.17 | 12.12 | 0.676 | n/a | n/a | n/a |
| Left Carpal (LEB-LCP-MC) | 90.87 | 10.35 | 0.211 | 0.10 | 0.446 | 94.63 | 7.35 | 82.86 | 6.65 | 93.73 | 10.82 | 0.020 | 0.111 | 1.000 | 0.024 |
| Right Carpal (LEB-LCP-MC) | 89.94 | 15.90 | 0.654 | 0.19 | 0.156 | 99.89 | 12.59 | 75.56 | 9.78 | 94.98 | 13.31 | 0.003 | 0.012 | 1.000 | 0.009 |

**(Continued S7): Trotting kinematics**

| **Range of motion: flexion/ extension angle** | **Whole group** | | **Normality p-value** | **Correlation with slope** | | **Levelled** | | **Intermediate** | | **Sloped** | | **KW**  **p-value** | **Dunn posthoc** | | |
| --- | --- | --- | --- | --- | --- | --- | --- | --- | --- | --- | --- | --- | --- | --- | --- |
|  | **Mean** | **SD** |  | **r** | **p-value** | **Mean** | **SD** | **Mean** | **SD** | **Mean** | **SD** |  | **Le-In** | **Le-Sl** | **In-Sl** |
| Neck (Head-with-T8) | 13.36 | 4.07 | 0.209 | 0.36 | 0.005 | 11.70 | 2.83 | 11.25 | 3.09 | 14.98 | 4.36 | 0.090 | n/a | n/a | n/a |
| Mid Thorax (With-T8-L1) | 12.28 | 3.98 | 0.518 | 0.09 | 0.510 | 12.54 | 1.90 | 12.05 | 3.07 | 12.28 | 5.01 | 0.878 | n/a | n/a | n/a |
| Thoraco-lumbar (T8-L1-L5) | 10.71 | 2.82 | 0.557 | -0.08 | 0.560 | 11.33 | 0.88 | 11.72 | 2.93 | 9.94 | 3.20 | 0.347 | n/a | n/a | n/a |
| Lumbo-sacral (L1-L5-Sacr) | 6.64 | 1.90 | 0.255 | 0.01 | 0.927 | 6.42 | 1.49 | 6.94 | 0.58 | 6.58 | 2.45 | 0.612 | n/a | n/a | n/a |
| Tail set (L5-Sacr-Co3) | 6.65 | 1.83 | 0.402 | 0.25 | 0.051 | 6.44 | 2.47 | 5.62 | 1.35 | 7.29 | 1.60 | 0.089 | n/a | n/a | n/a |
| Co3 (Sacr-Co3-Co8) | 12.30 | 3.70 | 0.381 | -0.36 | 0.004 | 14.77 | 3.93 | 12.02 | 3.95 | 11.46 | 3.26 | 0.247 | n/a | n/a | n/a |
| Co8 (Co3-Co8-Co14) | 16.20 | 7.20 | 0.008 | -0.22 | 0.096 | 20.60 | 6.99 | 14.67 | 8.03 | 15.66 | 6.65 | 0.199 | n/a | n/a | n/a |
| Left Hip (IW-GT-LSF) | 45.46 | 6.78 | 0.724 | 0.23 | 0.073 | 43.08 | 4.85 | 46.40 | 9.73 | 45.74 | 5.66 | 0.649 | n/a | n/a | n/a |
| Right Hip (IW-GT-LSF) | 45.55 | 6.07 | 0.195 | 0.22 | 0.085 | 44.19 | 7.56 | 45.39 | 7.38 | 46.23 | 4.88 | 0.939 | n/a | n/a | n/a |
| Left Stifle (GT-LSF-LHK) | 61.91 | 4.37 | 0.370 | 0.02 | 0.861 | 59.98 | 2.81 | 65.52 | 3.88 | 60.52 | 4.00 | 0.018 | 0.061 | 1.000 | 0.030 |
| Right Stifle (GT-LSF-LHK) | 64.06 | 6.16 | 0.303 | 0.36 | 0.005 | 60.29 | 4.99 | 64.86 | 8.01 | 65.25 | 4.80 | 0.319 | n/a | n/a | n/a |
| Left Hock (LSF-LHK-MT) | 62.62 | 7.43 | 0.149 | 0.05 | 0.708 | 62.40 | 9.02 | 63.64 | 5.79 | 62.16 | 7.99 | 0.886 | n/a | n/a | n/a |
| Right Hock (LSF-LHK-MT) | 64.77 | 10.55 | 0.535 | 0.37 | 0.004 | 59.88 | 8.55 | 62.05 | 13.07 | 68.62 | 8.63 | 0.113 | n/a | n/a | n/a |
| Left Shoulder (TS-AA-LEB) | 28.94 | 2.94 | 0.070 | 0.24 | 0.062 | 28.52 | 3.22 | 28.45 | 2.31 | 29.78 | 3.56 | 0.602 | n/a | n/a | n/a |
| Right Shoulder (TS-AA-LEB) | 36.03 | 3.06 | 0.685 | 0.30 | 0.021 | 34.43 | 3.71 | 37.74 | 2.67 | 35.95 | 2.63 | 0.492 | n/a | n/a | n/a |
| Left Elbow (HH-LEB-LCP) | 67.25 | 5.28 | 0.446 | 0.40 | 0.002 | 63.74 | 0.88 | 67.06 | 4.42 | 69.14 | 6.32 | 0.011 | 0.088 | 0.009 | 1.000 |
| Right Elbow (HH-LEB-LCP) | 66.36 | 6.64 | 0.023 | 0.27 | 0.039 | 64.63 | 5.92 | 67.90 | 5.17 | 66.15 | 8.44 | 0.575 | n/a | n/a | n/a |
| Left Carpal (LEB-LCP-MC) | 142.27 | 12.66 | 0.962 | 0.29 | 0.023 | 138.31 | 10.31 | 136.52 | 11.47 | 146.66 | 12.95 | 0.139 | n/a | n/a | n/a |
| Right Carpal (LEB-LCP-MC) | 141.03 | 13.26 | 0.995 | 0.11 | 0.390 | 143.81 | 13.73 | 134.50 | 14.58 | 143.77 | 11.73 | 0.326 | n/a | n/a | n/a |

**(Continued S7): Trotting kinematics**

| **Maximum protraction (negative) angle and maximum retraction (positive) angle (°)** | **Whole group** | | **Normality p-value** | **Correlation with slope** | | **Levelled** | | **Intermediate** | | **Sloped** | | **KW**  **p-value** | **Dunn posthoc** | | |
| --- | --- | --- | --- | --- | --- | --- | --- | --- | --- | --- | --- | --- | --- | --- | --- |
|  | **Mean** | **SD** |  | **r** | **p-value** | **Mean** | **SD** | **Mean** | **SD** | **Mean** | **SD** |  | **Le-In** | **Le-Sl** | **In-Sl** |
| **Maximum protraction (negative) angle** | | | | | | | | | | | | | | | |
| FL | -28.98 | 3.42 | 0.530 | -0.34 | 0.007 | -25.74 | 2.50 | -28.99 | 4.04 | -29.66 | 3.05 | 0.217 | n/a | n/a | n/a |
| FR | -29.38 | 4.33 | 0.492 | -0.37 | 0.004 | -26.25 | 6.34 | -29.48 | 2.98 | -30.59 | 3.61 | 0.263 | n/a | n/a | n/a |
| HL | -23.16 | 3.16 | 0.907 | 0.46 | <0.001 | -26.01 | 2.50 | -22.83 | 4.09 | -22.51 | 2.50 | 0.093 | n/a | n/a | n/a |
| HR | -23.03 | 3.65 | 0.342 | 0.22 | 0.095 | -25.06 | 4.46 | -22.31 | 5.05 | -22.81 | 2.58 | 0.459 | n/a | n/a | n/a |
| **Maximum retraction (positive) angle** | | | | | | | | | | | | | | | |
| FL | 40.24 | 2.93 | 0.918 | -0.12 | 0.354 | 41.26 | 3.54 | 39.70 | 2.12 | 40.30 | 3.28 | 0.622 | n/a | n/a | n/a |
| FR | 40.23 | 3.43 | 0.731 | -0.23 | 0.079 | 42.03 | 3.01 | 39.67 | 3.55 | 39.73 | 3.62 | 0.438 | n/a | n/a | n/a |
| HL | 36.81 | 2.70 | 0.607 | 0.39 | 0.002 | 35.27 | 3.93 | 36.46 | 1.81 | 37.42 | 2.68 | 0.425 | n/a | n/a | n/a |
| HR | 36.18 | 3.33 | 0.321 | 0.29 | 0.027 | 34.68 | 2.59 | 35.62 | 1.90 | 36.89 | 3.98 | 0.216 | n/a | n/a | n/a |
| **Range of motion: protraction/ retraction angle** | | | | | | | | | | | | | | | |
| FL | 69.22 | 3.92 | 0.201 | 0.21 | 0.107 | 67.00 | 1.44 | 68.69 | 4.36 | 69.96 | 4.03 | 0.581 | n/a | n/a | n/a |
| FR | 69.61 | 4.19 | 0.869 | 0.19 | 0.144 | 68.28 | 3.77 | 69.15 | 6.08 | 70.32 | 3.83 | 0.675 | n/a | n/a | n/a |
| HL | 59.97 | 3.46 | 0.992 | -0.11 | 0.403 | 61.29 | 4.72 | 59.29 | 4.61 | 59.93 | 2.51 | 0.652 | n/a | n/a | n/a |
| HR | 59.21 | 4.45 | 0.583 | 0.04 | 0.790 | 59.74 | 5.33 | 57.92 | 6.26 | 59.70 | 3.27 | 0.477 | n/a | n/a | n/a |

**(Continued S7): Trotting kinematics**

Kinematic abduction/ adduction joint angles recorded during trot (mean and inter-subject standard deviations) of the maximum and minimum joint angles for the breed and each slope group. Normality p-values show if parameters are normally distributed (p>0.05). The correlation coefficient (r) and p-values show the correlation between the parameter and slope of the back (significant correlation highlighted (p<0.05)). When Kruskal-Wallis (KW) p-values were significant, Dunn posthoc values were calculated between the groups: levelled (Le), intermediate (In) and sloped (Sl). Values are rounded to two decimal places. The anatomical markers used to calculate each joint angle in the Sagittal plane are shown in brackets.

|  | **Whole group** | | **Normality p-value** | **Correlation with slope** | | **Levelled** | | **Intermediate** | | **Sloped** | | **KW**  **p-value** | **Dunn posthoc** | | |
| --- | --- | --- | --- | --- | --- | --- | --- | --- | --- | --- | --- | --- | --- | --- | --- |
|  | **Mean** | **SD** |  | **r** | **p-value** | **Mean** | **SD** | **Mean** | **SD** | **Mean** | **SD** |  | **Le-In** | **Le-Sl** | **In-Sl** |
| **Minimum abduction (positive) angle or maximum adduction (negative) angle** | | | | | | | | | | | | | | | |
| Left Hip (IW-GT-LSF) | 21.70 | 4.20 | 0.874 | -0.06 | 0.755 | 21.69 | 4.92 | 22.38 | 3.68 | 21.38 | 4.39 | 0.870 | n/a | n/a | n/a |
| Right Hip (IW-GT-LSF) | 22.73 | 5.99 | 0.847 | -0.39 | 0.031 | 25.34 | 2.76 | 23.86 | 6.88 | 21.33 | 6.07 | 0.294 | n/a | n/a | n/a |
| Left Stifle (GT-LSF-LHK) | -3.42 | 5.16 | 0.718 | -0.16 | 0.411 | -0.19 | 5.78 | -4.38 | 5.18 | -4.24 | 4.63 | 0.318 | n/a | n/a | n/a |
| Right Stifle (GT-LSF-LHK) | -4.09 | 6.20 | 0.212 | 0.32 | 0.085 | -8.79 | 3.57 | -4.25 | 5.51 | -1.40 | 6.65 | 0.032 | 0.207 | 0.027 | 1.000 |
| Left Hock (LSF-LHK-MT) | 5.49 | 6.89 | 0.033 | -0.25 | 0.177 | 6.51 | 6.99 | 6.01 | 3.70 | 4.66 | 8.71 | 0.304 | n/a | n/a | n/a |
| Right Hock (LSF-LHK-MT) | 6.81 | 11.12 | 0.060 | -0.56 | 0.001 | 21.22 | 11.16 | 4.89 | 5.29 | 0.51 | 7.28 | 0.002 | 0.035 | 0.001 | 0.895 |
| **Maximum abduction (positive) angle and minimum adduction (negative) angle** | | | | | | | | | | | | | | | |
| Left Hip (IW-GT-LSF) | 39.60 | 4.18 | 0.777 | 0.08 | 0.677 | 38.83 | 4.35 | 40.66 | 4.23 | 39.42 | 4.30 | 0.688 | n/a | n/a | n/a |
| Right Hip (IW-GT-LSF) | 40.60 | 6.89 | 0.601 | 0.09 | 0.633 | 36.84 | 7.06 | 42.54 | 7.69 | 40.96 | 6.13 | 0.303 | n/a | n/a | n/a |
| Left Stifle (GT-LSF-LHK) | -46.15 | 15.24 | 0.102 | -0.28 | 0.139 | -38.54 | 20.75 | -47.87 | 14.17 | -48.30 | 13.31 | 0.459 | n/a | n/a | n/a |
| Right Stifle (GT-LSF-LHK) | -37.32 | 16.06 | 0.600 | -0.49 | 0.005 | -30.32 | 17.51 | -33.13 | 11.87 | -43.12 | 16.63 | 0.240 | n/a | n/a | n/a |
| Left Hock (LSF-LHK-MT) | 50.63 | 14.42 | 0.048 | 0.08 | 0.665 | 48.57 | 20.10 | 53.14 | 10.05 | 49.85 | 14.98 | 0.934 | n/a | n/a | n/a |
| Right Hock (LSF-LHK-MT) | 43.61 | 13.74 | 0.274 | 0.27 | 0.142 | 44.87 | 14.95 | 39.42 | 10.23 | 45.61 | 15.42 | 0.815 | n/a | n/a | n/a |

**(Continued S7): Trotting kinematics**

|  | **Whole group** | | **Normality p-value** | **Correlation with slope** | | **Levelled** | | **Intermediate** | | **Sloped** | | **KW**  **p-value** | **Dunn posthoc** | | |
| --- | --- | --- | --- | --- | --- | --- | --- | --- | --- | --- | --- | --- | --- | --- | --- |
|  | **Mean** | **SD** |  | **r** | **p-value** | **Mean** | **SD** | **Mean** | **SD** | **Mean** | **SD** |  | **Le-In** | **Le-Sl** | **In-Sl** |
| **Range of motion: abduction/ adduction angle** | | | | | | | | | | | | | | | |
| Left Hip (IW-GT-LSF) | 17.91 | 2.87 | 0.794 | 0.20 | 0.282 | 17.14 | 0.99 | 18.28 | 3.57 | 18.04 | 3.12 | 0.568 | n/a | n/a | n/a |
| Right Hip (IW-GT-LSF) | 19.38 | 3.98 | 0.330 | 0.37 | 0.045 | 15.84 | 0.30 | 20.70 | 3.54 | 19.63 | 4.33 | 0.052 | n/a | n/a | n/a |
| Left Stifle (GT-LSF-LHK) | 43.22 | 15.57 | 0.494 | 0.26 | 0.168 | 38.35 | 21.52 | 43.49 | 15.58 | 45.29 | 13.18 | 0.654 | n/a | n/a | n/a |
| Right Stifle (GT-LSF-LHK) | 34.43 | 16.95 | 0.988 | 0.65 | <0.001 | 21.53 | 17.81 | 29.23 | 11.91 | 45.24 | 13.47 | 0.014 | 1.000 | 0.021 | 0.117 |
| Left Hock (LSF-LHK-MT) | 45.89 | 13.89 | 0.850 | 0.27 | 0.144 | 42.06 | 20.26 | 47.13 | 11.98 | 46.88 | 12.45 | 0.737 | n/a | n/a | n/a |
| Right Hock (LSF-LHK-MT) | 38.04 | 14.30 | 0.081 | 0.76 | <0.001 | 23.65 | 3.99 | 35.33 | 9.92 | 47.85 | 13.33 | 0.002 | 0.123 | 0.001 | 0.367 |

**(Continued S7): Trotting kinematics**

Kinematic internal/ external rotations recorded during trot (mean and inter-subject standard deviations) of the maximum internal and external rotations for the breed and each slope group. Normality p-values show if parameters are normally distributed (p>0.05). The correlation coefficient (r) and p-values show the correlation between the parameter and slope of the back (significant correlation highlighted (p<0.05)). When Kruskal-Wallis (KW) p-values were significant, Dunn posthoc values were calculated between the groups: levelled (Le), intermediate (In) and sloped (Sl). Values are rounded to two decimal places. The proximal and distal segments of each joint were tracked using their clusters.

|  | **Whole group** | | **Normality p-value** | **Correlation with slope** | | **Levelled** | | **Intermediate** | | **Sloped** | | **Fr**  **p-value** | **Dunn posthoc** | | |
| --- | --- | --- | --- | --- | --- | --- | --- | --- | --- | --- | --- | --- | --- | --- | --- |
|  | **Mean** | **SD** |  | **r** | **p-value** | **Mean** | **SD** | **Mean** | **SD** | **Mean** | **SD** |  | **Le-In** | **Le-Sl** | **In-Sl** |
| **Maximum internal rotation (positive) or minimum external (negative) angle; right (positive) and left (negative) for back angles** | | | | | | | | | | | | | | | |
| Left Hip | 63.34 | 22.94 | 0.652 | -0.14 | 0.471 | 68.25 | 25.26 | 66.11 | 22.83 | 59.72 | 23.13 | 0.465 | n/a | n/a | n/a |
| Right Hip | 67.06 | 16.74 | 0.568 | -0.26 | 0.161 | 73.70 | 19.14 | 66.10 | 13.68 | 64.98 | 17.85 | 0.535 | n/a | n/a | n/a |
| Left Stifle | 35.48 | 13.79 | 0.281 | -0.02 | 0.906 | 30.17 | 10.00 | 44.00 | 15.71 | 32.49 | 12.19 | 0.140 | n/a | n/a | n/a |
| Right Stifle | 26.14 | 9.03 | 0.419 | 0.22 | 0.235 | 20.89 | 5.55 | 26.19 | 8.79 | 28.52 | 9.96 | 0.256 | n/a | n/a | n/a |
| Left Hock | -14.93 | 9.47 | 0.032 | 0.30 | 0.113 | -13.49 | 7.25 | -22.03 | 4.47 | -11.25 | 10.39 | 0.012 | 0.131 | 1.000 | 0.011 |
| Right Hock | -15.09 | 10.54 | 0.131 | 0.35 | 0.056 | -20.80 | 2.62 | -17.49 | 8.20 | -11.75 | 12.43 | 0.162 | n/a | n/a | n/a |
| Withers | 23.40 | 16.13 | 0.456 | -0.40 | 0.029 | 33.02 | 13.95 | 26.62 | 18.53 | 17.97 | 14.40 | 0.236 | n/a | n/a | n/a |
| Thoracic | 12.11 | 17.16 | 0.242 | 0.10 | 0.611 | 11.25 | 18.98 | 7.61 | 14.24 | 14.72 | 18.38 | 0.509 | n/a | n/a | n/a |
| Thoraco-lumbar | 12.74 | 12.20 | 0.250 | -0.09 | 0.638 | 18.97 | 13.16 | 10.69 | 5.93 | 11.76 | 14.24 | 0.299 | n/a | n/a | n/a |
| Lumbo-sacral | 11.17 | 11.67 | 0.154 | 0.36 | 0.053 | 3.38 | 11.99 | 13.17 | 7.84 | 13.22 | 12.54 | 0.187 | n/a | n/a | n/a |

**(Continued S7): Trotting kinematics**

|  | **Whole group** | | **Normality p-value** | **Correlation with slope** | | **Levelled** | | **Intermediate** | | **Sloped** | | **KW**  **p-value** | **Dunn posthoc** | | |
| --- | --- | --- | --- | --- | --- | --- | --- | --- | --- | --- | --- | --- | --- | --- | --- |
|  | **Mean** | **SD** |  | **r** | **p-value** | **Mean** | **SD** | **Mean** | **SD** | **Mean** | **SD** |  | **Le-In** | **Le-Sl** | **In-Sl** |
| **Maximum external (negative) angle or minimum internal (positive) angle; right (positive) and left (negative) for back angles** | | | | | | | | | | | | | | | |
| Left Hip | -32.82 | 21.09 | 0.294 | -0.25 | 0.182 | -30.54 | 18.11 | -28.27 | 23.87 | -36.46 | 21.19 | 0.717 | n/a | n/a | n/a |
| Right Hip | -20.66 | 15.97 | 0.191 | -0.16 | 0.392 | -13.57 | 9.33 | -22.91 | 14.93 | -22.28 | 18.44 | 0.506 | n/a | n/a | n/a |
| Left Stifle | -19.00 | 15.96 | 0.078 | -0.23 | 0.225 | -16.60 | 9.89 | -15.31 | 18.60 | -22.14 | 16.85 | 0.565 | n/a | n/a | n/a |
| Right Stifle | -20.83 | 11.25 | 0.017 | 0.24 | 0.207 | -26.28 | 14.63 | -16.53 | 6.12 | -21.26 | 11.86 | 0.382 | n/a | n/a | n/a |
| Left Hock | -40.36 | 10.32 | 0.941 | 0.16 | 0.405 | -37.19 | 5.61 | -47.25 | 6.87 | -37.49 | 11.78 | 0.023 | 0.095 | 1.000 | 0.033 |
| Right Hock | -39.71 | 9.07 | 0.402 | 0.20 | 0.278 | -40.98 | 3.83 | -41.45 | 10.76 | -38.01 | 9.49 | 0.742 | n/a | n/a | n/a |
| Withers | -28.05 | 20.53 | 0.784 | -0.52 | 0.003 | -16.59 | 17.01 | -23.87 | 15.31 | -35.91 | 22.62 | 0.112 | n/a | n/a | n/a |
| Thoracic | -18.09 | 18.48 | 0.266 | 0.11 | 0.556 | -23.75 | 19.21 | -17.31 | 10.86 | -16.57 | 21.54 | 0.701 | n/a | n/a | n/a |
| Thoraco-lumbar | -10.05 | 13.24 | 0.664 | 0.13 | 0.489 | -13.62 | 15.47 | -9.35 | 6.89 | -8.99 | 15.31 | 0.846 | n/a | n/a | n/a |
| Lumbo-sacral | -9.62 | 15.30 | 0.377 | 0.27 | 0.156 | -18.16 | 6.87 | -7.67 | 16.19 | -7.37 | 16.71 | 0.074 | n/a | n/a | n/a |
| **Range of motion: internal/ external rotation angle** | | | | | | | | | | | | | | | |
| Left Hip | 96.16 | 19.53 | 0.652 | 0.11 | 0.564 | 98.79 | 9.30 | 94.38 | 18.07 | 94.68 | 23.97 | 0.767 | n/a | n/a | n/a |
| Right Hip | 87.03 | 20.20 | 0.279 | -0.08 | 0.663 | 87.28 | 26.76 | 86.47 | 16.42 | 84.81 | 19.27 | 0.998 | n/a | n/a | n/a |
| Left Stifle | 53.21 | 15.15 | 0.566 | 0.18 | 0.352 | 46.76 | 3.54 | 57.61 | 13.12 | 51.89 | 18.52 | 0.117 | n/a | n/a | n/a |
| Right Stifle | 44.75 | 16.42 | 0.130 | -0.11 | 0.551 | 47.18 | 18.78 | 42.73 | 11.10 | 47.78 | 17.28 | 0.775 | n/a | n/a | n/a |
| Left Hock | 24.61 | 7.66 | 0.463 | 0.09 | 0.621 | 23.70 | 4.91 | 25.23 | 7.85 | 24.05 | 8.96 | 0.893 | n/a | n/a | n/a |
| Right Hock | 21.61 | 16.11 | 0.245 | 0.03 | 0.881 | 20.18 | 3.08 | 23.96 | 6.23 | 24.33 | 18.41 | 0.426 | n/a | n/a | n/a |
